# Patterns of allele frequency differences among domestic cat breeds assessed by a 63K SNP array

**DOI:** 10.1371/journal.pone.0247092

**Published:** 2021-02-25

**Authors:** Hasan Alhaddad, Mona Abdi, Leslie A. Lyons

**Affiliations:** 1 Department of Biological Sciences, Kuwait University, Safat, Kuwait; 2 Department of Veterinary Medicine and Surgery, College of Veterinary Medicine, University of Missouri - Columbia, Columbia, Missouri, United States of America; HudsonAlpha Institute for Biotechnology, UNITED STATES

## Abstract

Cats are ubiquitous companion animals that have been keenly associated with humans for thousands of years and only recently have been intentionally bred for aesthetically appealing coat looks and body forms. The intense selection on single gene phenotypes and the various breeding histories of cat breeds have left different marks on the genomes. Using a previously published 63K Feline SNP array dataset of twenty-six cat breeds, this study utilized a genetic differentiation-based method (*di*) to empirically identify candidate regions under selection. Defined as three or more overlapping (500Kb) windows of high levels of population differentiation, we identified a total of 205 candidate regions under selection across cat breeds with an average of 6 candidate regions per breed and an average size of 1.5 Mb per candidate region. Using the combined size of candidate regions of each breed, we conservatively estimate that a minimum of ~ 0.1–0.7% of the autosomal genome is potentially under selection in cats. As positive controls and tests of our methodology, we explored the candidate regions of known breed-defining genes (e.g., *FGF5* for longhaired breeds) and we were able to detect the genes within candidate regions, each in its corresponding breed. For breed specific exploration of candidate regions under selection, eleven representative candidate regions were found to encompass potential candidate genes for several phenotypes such as brachycephaly of Persian (*DLX6*, *DLX5*, *DLX2*), curled ears of American Curl (*MCRIP2*, *PBX1)*, and body-form of Siamese and Oriental (*ADGRD1*), which encourages further molecular investigations. The current assessment of the candidate regions under selection is empiric and detailed analyses are needed to rigorously disentangle effects of demography and population structure from artificial selection.

## Introduction

The cat, *Felis catus*, was likely domesticated around 10,000 years ago but only recently has imposed artificial selection created pedigreed cats that are generically referred to as cat breeds [1–3]. Since the first cat shows held in London’s Crystal Palace in 1871 and later in New York’s Madison Square Garden in 1881, many new breeds have been developed [4–6]. Currently, 40–71 breeds are recognized by different cat fancy organizations (Cat Fanciers’ Association (CFA)—42 [7], The International Cat Association (TICA)—71 [8], Governing Council of the Cat Fancy (GCCF)—40 [9], and Federation Internationale Feline (FIFE)—48 [10] breeds). The difference in the number of recognized breeds is related to the different criteria used to define a breed. Unlike other domesticated animals, which were selected for physical, behavioral, production, or functional traits and resulted in extreme breed variation [11–15], cat breeds were mostly selected for discrete, single gene traits that were aesthetically pleasing to breeders [16].

In general, cat selected phenotypes can be classified into two groups; one related to the coat (color, pattern, length and texture) and a second related to body morphology (face, ear, body size, legs and tail) [17]. Most of the external variation, especially those that define breeds, can be seen in the coat and less so in body morphology [16, 18]. Cat aesthetic traits differ in (1) type; coat, body variations, or combinations, (2) origin; recently propagated *de novo* mutation or selected from natural populations, (3) history; an old trait or recently discovered, (4) specificity; trait present in a single breed or shared amongst multiple breeds. These differences between the selected traits are likely to have different impacts on the genomes of various breeds.

Cat coat color traits belong to two groups; (1) patterned, which is displayed as a combination of more than one color in the form of spotted, striped, swirls and dorsal-ventral pigmentation, or (2) pigmentation, where colors range from white to heavily pigmented color. Cat coat color phenotypes are products of genetic variation within *KIT* (white and white spotting) [19–21], *TYR* (points pattern, mocha, albino) [22–24], *ASIP* (black, agouti) [25], *MC1R* (amber and russet) [26, 27], *TYRP1* (brown, cinnamon and chocolate) [28], and *MLPH* (dilute coloration) [29]. Similarly, coat length and texture arose from polymorphisms within *FGF5* (longhair) [30, 31], *KRT71* (curly and naked) [32, 33], and *LPAR6* and *LIPH* (curly) [34, 35]. On the other hand, body morphology variations that were selected in various breeds include the face (Burmese—*ALX1*) [36, 37], ears (Scottish Fold folded ears—*TRPV4*) [38], size (dwarfism—*UGDH*) [39], and tail (Japanese Bobtail tail length and kink—*HES*7, Manx tail length and tailless–*T* gene) [40–42].

The origin of cat breed defining traits generally followed one of three paths [43]. One path depended on selecting individual cats from the standing variation within a feral population. Breeds developed by this route are referred to as natural breeds (i.e. Persian, Turkish Angora, Maine Coon and Siberian) [44]. A second relied on combining traits of two breeds or two cat species and develop a cross-hybrid. The Ocicat’s defining look was developed using other established breeds (Abyssinian, American Shorthair and Siamese). The Bengal breed is a hybrid between domestic cats and the Asian leopard cat, *Prionailurus bengalensis* [6]. A third way was to identify a *de novo* mutation (i.e. an individual with unique trait) and carefully perform selective matings to fix and propagate the newly discovered phenotype (e.g., Selkirk Rex [45]).

Recent and rapid increase in cat breeds through strong selection directed for desired single traits and crossing of unrelated pre-existing breeds, created a bulk of interrelated and yet phenotypically divergent breeds. However, the majority of breeds were established by selecting from feral populations (new phenotype or pre-existing one) and can be traced to four regional populations: (1) Western derived (e.g. Persian, Main Coon, and Norwegian Forest Cat), (2) Eastern derived (e.g. Birman, Burmese, Bombay, Siamese and Oriental), (3) Mediterranean (Turkish Van and Turkish Angora), and (4) Arabian Sea (Sokoke) [16, 43, 44].

The development of high-throughput genotype arrays has permitted the possibility of identifying chromosomal regions and genes targeted by artificial selection in various domestic animals species (e.g. cats [46], dogs [47, 48], cattle, [11, 49, 50], and horse [15]). Correspondingly, the current study aimed to investigate the signatures of selection of twenty-six recognized cat breeds using 63K SNP array data and provide an overall representation of the candidate genomic regions across all breeds, which we refer to as the “landscape of selection”. The specific objectives included: (1) identifying candidate regions under selection for each breed, (2) evaluating the biological significance of the detected candidate regions using known phenotype-causing variants, (3) exploring the gene content within a representative number of candidate regions for likely phenotype association, and (4) inspecting candidate regions that are present in several closely related breeds.

## Results

### Information on selected dataset

Seven hundred thirty-seven cats representing 26 recognized cat breeds were included in the study (S1 Table). Seven breeds (Persian, Scottish Fold, Siberian, Bengal, Ragdoll, Birman and Burmese) were represented by 50 samples. To conduct an intraspecific comparison within a single breed, the samples of these seven over represented breeds were randomly divided into two groups (e.g., Bengal 1 and Bengal 2). The breed sample sizes ranged from 10 to 26 cats and twenty of the breeds were represented by 20 or more cats.

As a preparation of the workable SNP dataset, autosomal markers (n = 58,888) were extracted from the genotype dataset by removing the X-chromosome SNPs (n = 2,700) and the unmapped SNPs (n = 684). Approximately 99.7% (n = 58,768) of the autosomal markers passed SNP genotyping call rate of 90% (each SNP genotyped in at least 90% of the samples), thus, only 120 SNPs were excluded across all samples. The average percentage of monomorphic SNPs within each breed was 31.7% ranging from (19.1%–54.8%) (S1 Table). Approximately 12% SNPs (n = 6,955) had a MAF between (0 and 0.05) across all populations and were excluded. Overall, 50709 autosomal SNPs that were accurately mapped to the latest cat genome assembly (felCat 9.0 [51]) were included in downstream analyses.

**Fig 1:**
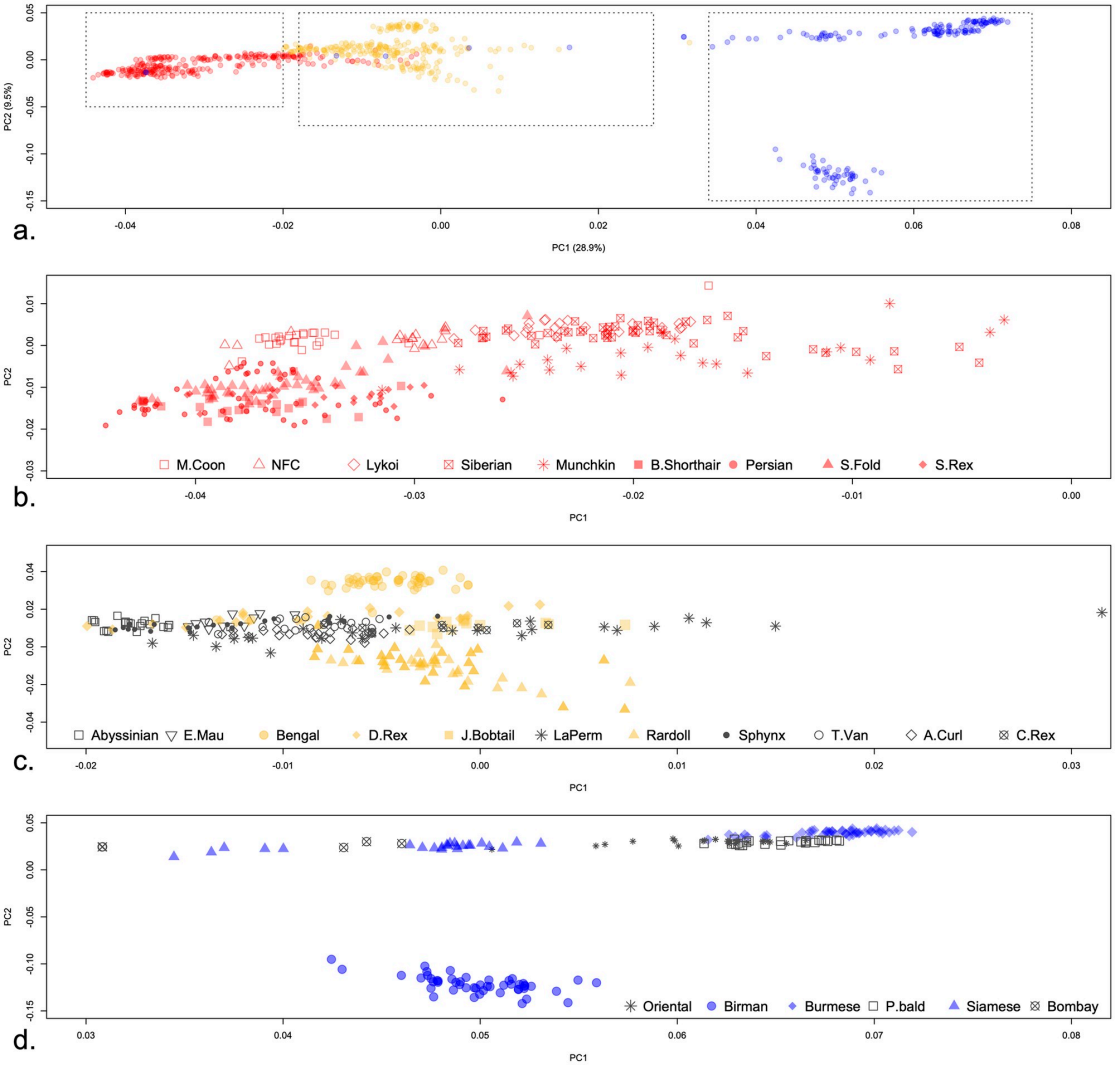
Population structure of cat breeds via principle component analysis (PCA). The first two principle components (PC1 and PC2) explain majority of the genetic variation among cat breeds. (a) A broad overview of the population structure of all breeds. Each individual cat is represented by a circle that is colored according to its ancestral group (Western-red, “Middle”-yellow, Eastern-blue) and dashed boxes are used to designate 9 Western breeds (left box), 11 intermediate breeds (middle box) and 6 Eastern breeds (left box). The blue dots extending to the “Middle” and Western groups belong to Bombay breed. Note that Bombay breed is formed by breeding of Burmese and American Shorthair. (b-d) Individual representations of Western, “Middle”, and Eastern breeds, respectively. Note the separation of the interspecies hybrid breed, Bengal, from other “Middle” breeds in (b) and separation of the phenotypically distinct Birman from other Eastern breeds in (c). Six Bombay individuals were outside of the ancestral grouping of Eastern breeds were removed in (d) for ease of presentation.

To understand the genetic relationships among the breeds under study, PCA was performed and the first two components PC1 and PC2 explained ~ 40% of the total genetic variation (Fig 1). A broad overview showed that the first component, PC1, which accounts for 28.9% of the total variation, structured the breeds according to their origin ancestry as a continuum from Western to Eastern (Fig 1a). Additionally, Western breeds and what we refer to as “Middle” breeds clustered more closely than to the Eastern breeds, which forms a distant distinct group. The population structure Western breeds can be summarized by: (1) the Persian family breeds (Persian, British Shorthair, Scottish Fold, and Selkirk Rex) formed a distinct group, (2) Maine Coon breed formed a single distinct group, and (3) individuals from the Munchkin and Siberian were less closely structured and extended to the “Middle” breeds (Fig 1b). The “Middle” breeds were less distinctly structured with the exception of Bengal, which formed a separate group (Fig 1c). Two general groups were detected in the Eastern breeds cluster where Birman separated from the rest (Fig 1d). Individuals of the Bombay breed did not form a unique group and some extended to the “Middle” and “Western” clusters (Fig 1a). The second component, PC2, that explains 9.5% of the total variation enabled separation of Bengal from other “Middle” breeds (Fig 1c) and Birman from Eastern breeds (Fig 1d).

### Selection statistical test (*di*)

As a calculation of likely signature of selection, SNP-specific *di* values were calculated for each breed. averaged for SNPs within individual overlapping 500Kb windows (50% overlap between adjacent windows). The 99^th^ percentile threshold (top 92 windows) were reported as candidate windows under selection (S1 Fig, S2 Table).

Collectively across breeds, individual candidate windows under selection (top 92) from each breed were distributed across the autosomal chromosomes (S1 Fig, S2 Table). This posed challenges such as: (1) despite being on different locations, the number of candidate windows (92) is identical for each breed, which prevents objective comparisons between breeds, (2) the likelihood of false positive windows due to SNP ascertainment bias associated with the design of the SNP array, (3) the uneven distribution of SNPs across windows, and (4) the lack of a systematic method that prioritizes certain candidate windows for gene content investigation. To overcome these challenges, we sought to identify candidate regions under selection rather than to explore individual scattered windows. We define a candidate region under selection as three or more overlapping candidate windows, which combined would be ≥ 1Mb in size (S3 Table).

Using the aforementioned approach, nearly 50% of the candidate windows in each breed were single scattered windows or only two overlapping windows (S2 Fig, S2 Table). The categorization of candidate windows is only for prioritizing which candidate regions to be investigated for gene contents. These candidate windows were not totally dismissed from subsequent analyses but later used when discussing specific breeds (e.g., Persian) or inspecting specific chromosomal locations (e.g., *FGF5* on Chr. B1). Abyssinian breed showed the lowest percentage of nonoverlapping candidate windows (50%) whereas Bombay had over 90% of its candidate windows as single or two windows (S2 Fig).

### Candidate regions of selection

**Fig 2:**
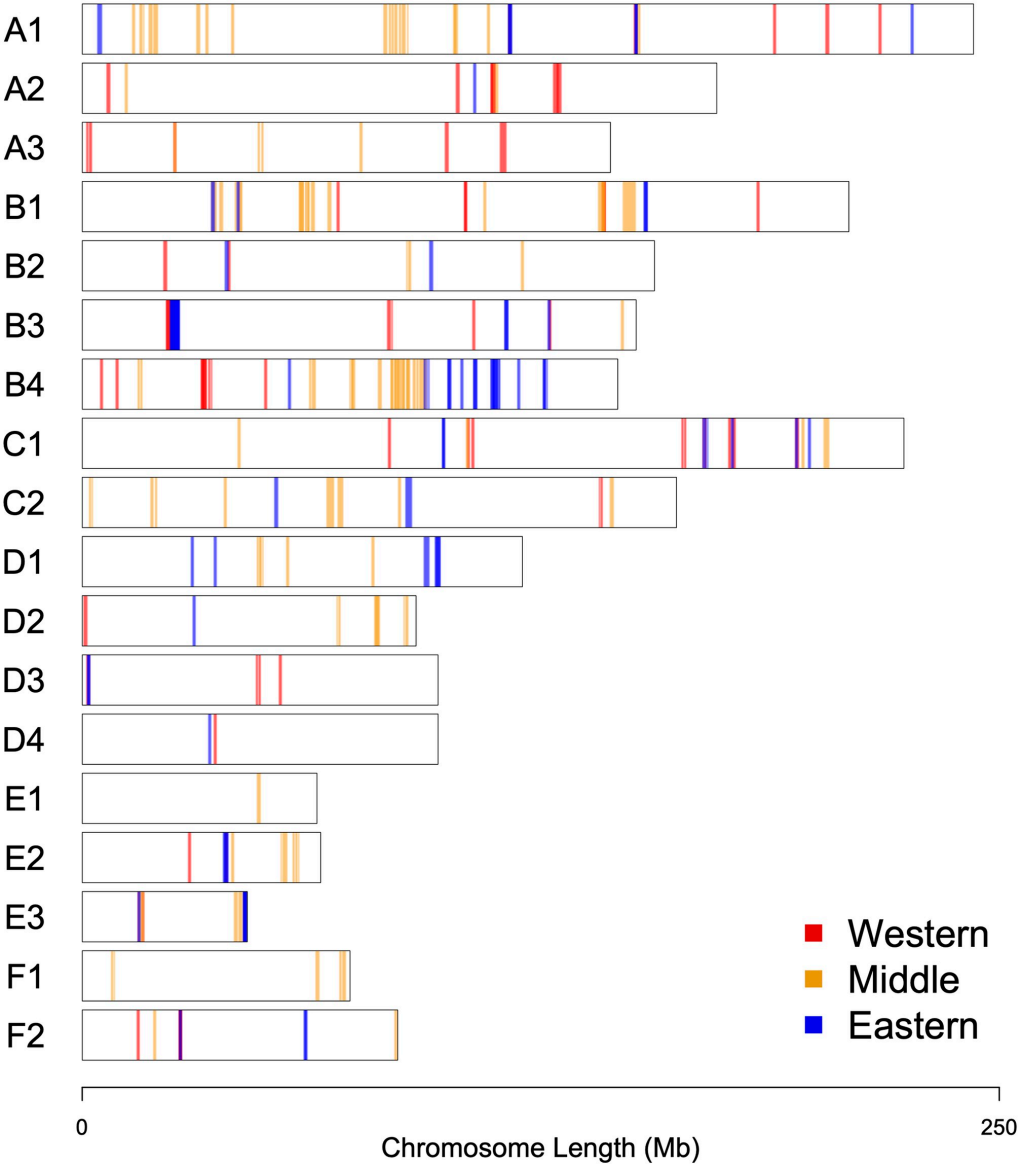
The genomic landscape of selection in domestic cat genome. Chromosomal representation of 205 candidate regions under selection across all cat breeds. Colored vertical bars represent candidate regions under selection and the intensity of color reflects the number of breeds having the particular candidate region. Red, yellow, and blue colored bars correspond to Western (9), “Middle” (11), and Eastern (6) breeds, respectively.

Combining and overlaying the identified candidate regions under selection for all breeds provides an overall look at the genomic landscape of selection in the pedigreed domestic cats (Fig 2). Using the 205 candidate regions (S3 Table) of 26 breeds (not accounting for redundancy), the following regarding the genomic landscape of selection in cats was observed: (1) candidate regions were found variably in all chromosomes (S3a Fig), (2) the largest number of candidate region were observed in Chrs. B4, A1, and B1, respectively whereas the least number were in Chrs. E1, D4, and F1 (S3a Fig), (3) the number of candidate regions were in correlation with the size of the chromosome with the exception of Chr. B4 and E3 (S3b Fig), (4) the majority of candidate regions were composed of three overlapping windows and measured 1Mb in size (S3c and S3d Fig), (5) the average size of candidate regions was 1.5 Mb, and the largest candidate region (10 Mb) was found on Chr. B4 (S1 Fig).

Whilst candidate regions expectedly varied in number and size between chromosomes, variation was also observed between breeds (S1 Table, S2 Fig). The number of candidate regions of selection averaged to ~ 6 regions per breed where the least was found in Bombay (two regions) and the most was in Birman (11 regions) (S3 Table). Similarly, the average combined size of candidate regions per breed was 9.3 Mb and ranged from 2.5 Mb in Bombay to a 16.25 Mb in Ragdoll (S1 Table, S4 Fig). When examining the number and the combined sizes of candidate regions per breed in relation to the ancestral groups (Western, “Middle”, Eastern), we observed that Eastern breeds (excluding Bombay) had larger number of candidate regions per breed (averaged ~ 9 regions) and covering larger regions (averaged ~ 12 Mb) compared to “Middle” (~ 6 regions and 10.4 Mb), and Western breeds (~ 5 regions and 7 Mb).

We adopted the exclusion of single or two windows regions as a strategy to prioritize the importance of candidate regions and to provide conservative estimates of candidate regions under selection for each breed. However, we find it more useful to include all candidate windows when comparing two populations of the same breed (e.g., Bengal 1 and Bengal 2) or between different breeds especially since partial shared windows are often observed (S1 Fig).

Intra-specific breed comparison was conducted on seven breeds representing the three ancestral groups (Western, “Middle”, and Eastern). The shared candidate windows within a breed ranged from 38% for Scottish Fold to over 84% for Burmese (S4 Table). The Western breeds (Persian, Scottish Fold, and Siberian) exhibited lower number of shred candidate windows among the subpopulations (38–53%) while Bengal and Ragdoll as representatives of “Middle” breeds shared (62–64%). Eastern breeds (Birman and Burmese) showed the highest number of shared windows (77–84%). The unique and unshared windows between the two populations within an individual breed were mostly of single scattered windows or two overlapping windows (S4 Table). This independently supports our strategy of defining candidate regions under selection by three or more overlapping windows.

Many individual windows were shared across several breeds (examples in S1 Fig Chrs. A1, B1, and B2). However, more significantly was the presence of shared candidate regions. One example of such regions was located on Chr.B3 and shared among Bombay, Burmese, Peterbald, Oriental, and Siamese and less uniformly with Scottish Fold and Siberian. A second region is located on Chr. E3 and is shared across seven breeds from the three ancestral groups (S1 Fig).

### Candidate regions as positive controls

Genetic variants that are associated with phenotypes under selection in cats serve as useful positive controls for our conducted analyses and a display of the validity subsequent reported findings. Our five positive controls were: (1) Cornish Rex and multiple candidate regions surrounding its curly coat causative gene, *LPAR6* (S1 Fig-ChrA1), (2) Abyssinian and a candidate region where *ASIP* resides, which explains the lack the non-agouti of its coat (S1 Fig-Chr.A3), (3) Persian, Norwegian Forest Cat, Siberian, and Turkish Van with candidate regions of different sizes around the longhair gene, *FGF5* (S1 Fig-Chr.B1), (4) Devon Rex’s candidate region for the curly coat associated gene, *KRT71* (S1 Fig-Chr.B4), and (5) Siamese a *TYR* gene candidate region, which is responsible for the distinct dark coat coloration at the extremities (S1 Fig-Chr.D1).

Dominantly inherited genetic variants, previously identified for breed-defining phenotypes, were also observed. The candidate regions encompassing or adjacent to such variants were mostly represented by single or two windows. Examples of these regions included: (1) windows surrounding *UGDH* gene, which is related to dwarfism in Munchkins (S1 Fig-Chr.B1), (2) a single window adjacent to *KRT71*, which is responsible for the curly coat of Selkirk Rex (S1 Fig-Chr.B4), and (3) a window covering the tail-length causative gene, *HES*, in Japanese Bobtail (S1 Fig-Chr.E1).

### Single breed candidate regions

We explored the candidate regions under selection for each breed and examined genes within for relationships to the breed defining phenotypes (S3 Table). However, we limited our discussion to a representative number of candidate regions selected based on their (1) sizes, (2) presence of phenotype related candidate genes, and when applicable (3) occurrence in replica populations (i.e., Persian1 and 2). Below, we provide detailed examples for three breeds while others are reported in Table 1.

**Table 1:** Representative candidate regions under selection in cat breeds with likely phenotype related candidate genes.

Breed	Chr.	Start (Mb)	End (Mb)	Region size (Mb)	No. genes	Candidate gene(s)	Phenotype
Perisan^1^	A2	101.75	103	1.25	8	*DLX6*, *DLX5*	Brachycephaly
	C1	163.25	164.75	1.5	6	*DLX2*	
Maine Coon	A1	188.25	189.25	1	4	*GABRG2*, *GABRA1*, *GABRA6*, *GABRB2*	Eating behavior/size
Bengal^1^	D2	79.5	81.25	1.75	13	*FGFR2*	Coat type
Turkish Van	E3	43.75	44.75	1	48	*CLCN7*^2^	Van color
American Curl	E3	41.25	44.75	3.75	123	*MCRIP2*	Curled ears
	F1	63.5	45	1.25	6	*PBX1*^3^	
La Perm	B1	147.25	151	3.75	26	*EPGN*, *EREG*	Curly coat
Ragdoll^1^	A1	82	89	7	40	*LAMP1*	Multiple
Birman^1^	B4	11.25	114	2.75	8	*KITLG*	Gloves
Oriental	D3	1.25	2.25	1	5	*ADGRD1*	Body weight
Siamese	D3	1.25	2.25	1	5	*ADGRD1*	Body weight

^1^ Candidate regions were found completely or partially shared among two populations (n = 25 cats each) of the same breed.

^2^ Due to the low density of SNPs on Chr.E3, flanking regions to the candidate region were explored. Candidate gene (*CLCN7*) is located upstream of the region at ~ 43.3Mb.

^3^ Due to low density of SNPs on Chr.F1, flanking regions were explored. Candidate gene (*PBX1*) is located upstream of the region at ~ 63Mb.

**Fig 3:**
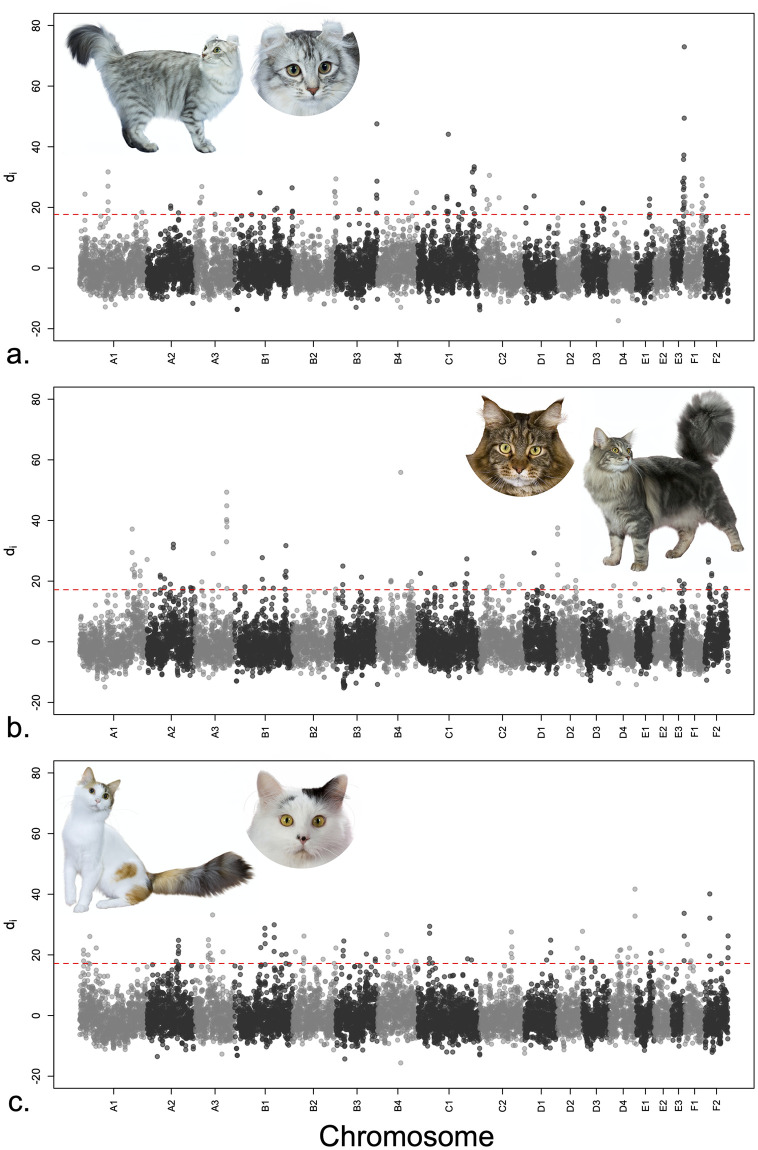
Manhattan plots of genome-wide (*di*) measures of overlapping 500Kb windows for three cat breeds. Each dot represents a window of 500 Kb (total 9108 windows) and the horizontal dashed red line indicates the 99^th^ percentile threshold, which distinguishes the top 92 windows. (a) Genome-wide survey of selection for the America Curls, which is a breed defined by backward curled ears. A number of overlapping windows clearly show a signal on Chr. E3. (b) Genome-wide overview of selection for Maine Coon cats, which is a breed recognized by an overall large size and facial features such large oval eyes and a square chin. Visible signals of overlapping windows are shown on Chrs. A1 and A3. (c) Signal of selection for the Turkish Van breed, which is acknowledged for a coat color pattern named after the breed, “Van”. This pattern appears in an overall white coat with distinct coloration in parts of the head and tail. Few overlapping windows were detected on Chrs. A2 and E3. Cat photographs were provided with permission by Larry Johnson.

Genome-wide analysis of the signatures of selection for the American Curl breed showed distinctly several overlapping windows with high levels of genetic population differentiation on Chr.E3 and F1 (Fig 3a). The size of the first candidate region was 3.75Mb and contained 123 genes among which *MCRIP2* representing a possible candidate gene for the backward curled ears. A second smaller candidate region was detected with *PBX1* as another candidate gene (Table 1). Similarly, Maine Coon’s genome-wide survey showed two apparent regions on Chrs.A1 and A3 (Fig 3b). The candidate region on Chr.A1 was a 1Mb region and contained four genes (*GABRG2*, *GABRA1*, *GABRA6*, *GABRB2*) whereas the region on Chr.A3 was 2Mb with two genes, *FAM98A* and *RASGRP3*. Lastly, with a focus on the unique coloration of the Turkish Van, which is characterized by an overall white coat with different coloration of parts of the head and tail, we identified two candidate regions (Fig 3c). The regions were located on Chrs.A2 and E3 with the latter harboring *CLCN7* as candidate genes, (Table 1).

### Common selected region across several breeds

**Fig 4:**
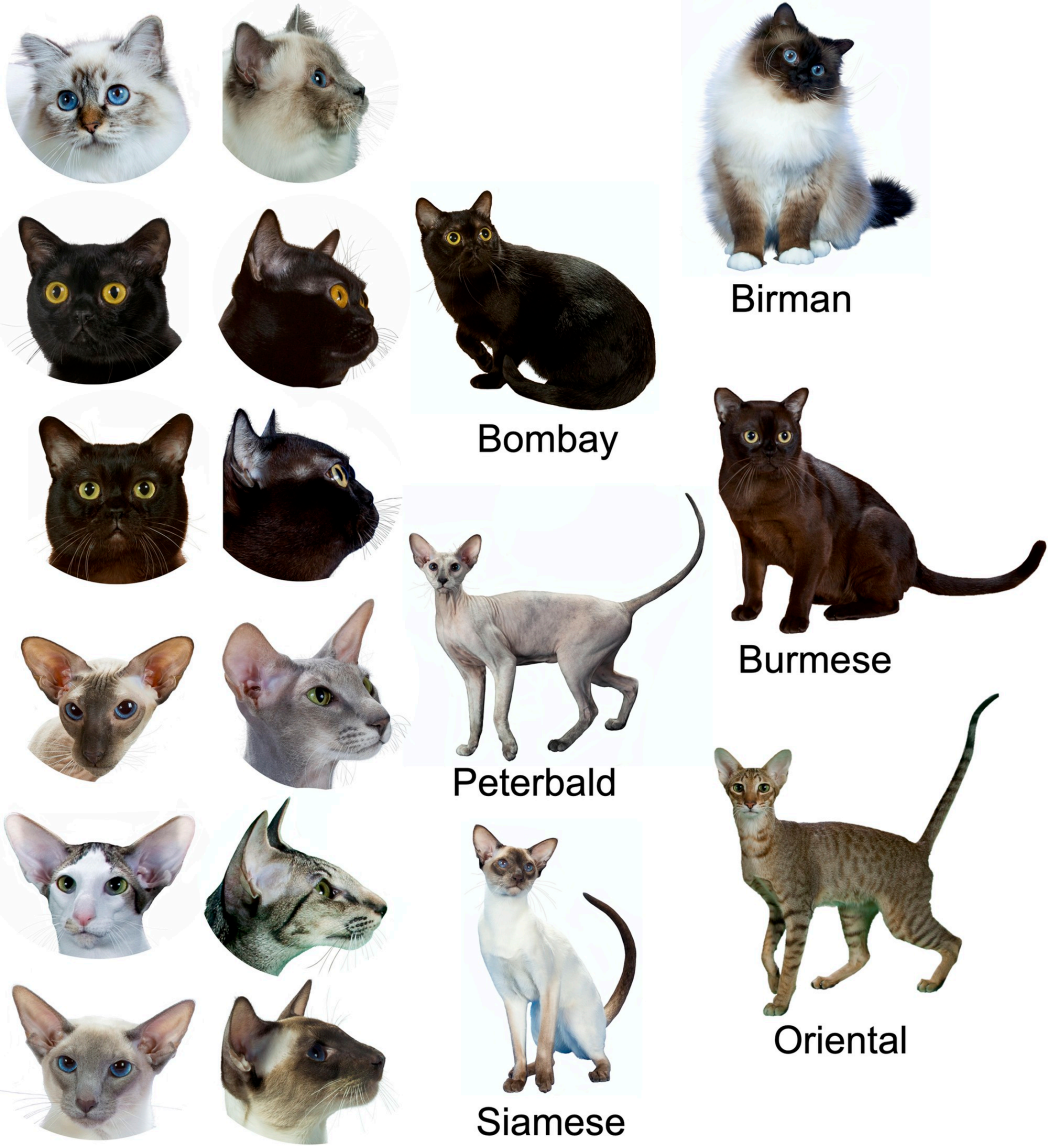
Morphological variation in body-form between six Eastern breeds. Birman symbolizes one extreme end of the physical spectrum of Eastern breeds by displaying stocky body, broad and round head, rounded muzzle, and round eyes whereas Siamese, Oriental, and Peterbald display the other extreme end through a tubular body, wedged head, wedged muzzle, and almond-shaped eyes. Burmese, Bombay exhibit intermediate body-shapes and facial features between the two extremes. Cat photographs were provided with permission by Larry Johnson.

A shared candidate region of relatively low SNP density was found in Turkish Van, American Curl, Japanese Bobtail, Peterbald, Oriental, and Siamese (S1 Fig, S3 Table). This shared candidate region among diverse breeds was located on Chr. E3 and contained 36 genes. A second shared candidate region was identified specifically in the breeds of slender body shape; Peterbald, Oriental, and Siamese (See Fig 4 for body shape). The Chr. D3 region was 1Mb in size and contained five genes among *ADGRD1* as potential candidate for the unique body form (Table 1).

## Discussion

Recent and rapid selection in the domestic cat has resulted in the formation of 40–71 cat breeds that are recognized today [7–10]. Selection in the cat focused on single or few aesthetic external traits, which are considered breed defining [16]. The distinct curly coat of Selkirk Rex [52] and the tail morphology of Japanese Bobtail [53] are examples of cat breed development practices. A different scenario is the Somali breed, which is only a longhair variant of Abyssinian cats, sharing nearly identical breed standards [7]. This study (1) explored the fingerprints of the cat selection history and breeding practices using genome-wide SNPs genotyped in 26 breeds, (2) highlighted candidate regions under selection, and (3) reported likely phenotype-related genes.

Despite the intense selection imposed during the formation of the cat breeds, only ~ 2.5–16.25 Mb appeared under selection, which constituted 0.1–0.7% of the autosomal genome. Using similar analytical approaches, the number of candidate regions identified for the cat (205 in 26 breeds) is smaller than the ones identified for the dog (275 in 10 breeds) [48], the horse (695 in breeds) [15], and the cow (583 in 5 breeds) [54]. The difference in number of candidate regions under selection between cat breeds and other domesticated animals may largely be due to limited selection on a single or few genes in cats whereas in other animals the selection is on quantitative trait loci (QTL).

### Cat breeds and signatures of selection

While top 1% candidate windows (n = 92) under selection for each breed were reported (S2 Table), we prioritized the candidate windows into candidate regions based on the presence of multiple overlapping windows in the same genomic location (S3 Table). This ensured reporting of conservative estimates of candidate regions under selection in each breed, allowed objective comparison between breeds, and avoided false positives due to ascertainment bias. On average, cat breeds had 2–12 regions under selection positioned on 2–8 chromosomes and with combined size of 2.5–16.25 Mb. The number and size of candidate regions varied among breeds based on: (1) ancestry (e.g., Western), (2) population size (i.e., popularity), (3) age of the breed-defining trait(s) (e.g., longhair), (4) mode of inheritance of the trait under selection, and (5) breeding practices (e.g., outcrossing). An example of the differences observed based on breed ancestry was the relative low number and small size of candidate regions under selection in Western breeds contrasted by the high number of large size candidate regions in Eastern breeds (S1 Table). This is consistent with previously reported differences in estimated population parameters such as linkage disequilibrium, genetic diversity, and levels of inbreeding [55, 56].

Persian, including Exotics as a short-haired variant, is the most popular and largest purebred cat population. The effect of population size of the Persian breed on the candidate region under selection was evident in the few small sized candidate regions (max = 1.5 Mb). This effect was especially apparent when comparing the two replica populations (Persian 1 and 2) where 50% of the candidate windows were shared (S4 Table). The variability in the detect candidate regions under selection mirrors the variability observed in its signature brachycephaly phenotype and the range of diseases [57]. Nonetheless, two shared candidate regions among the two Persian populations contained promising candidate genes for brachycephaly (Table 1). Both candidate regions contained distal-less homeobox genes (Chr.A2: *DLX6* and *DLX5*—Chr.C1: *DLX2*), which are involved in the craniofacial development [58, 59]. We suspect that several candidate genomic regions and several genes are responsible for the wide range of brachycephaly phenotypes in Persian cats.

A comparison between the Turkish Van and the Bengal breeds provides an illustration to the age the breed and its selected phenotypes on the numbers and sizes of candidate regions under selection. The Turkish Van is an old breed and belongs to a Mediterranean ancestral cluster [16, 44] with signature coat color pattern named after the breed, “Van”. This coat color appears as an overall white color with different colors randomly covering parts of the head and tail (Fig 3c). On the other hand, the Bengal breed is a comparatively younger breed that resulted from an interspecies hybridization [6] with a marking phenotype of patterned, soft, and glittered coat. Reminiscent of the old age of the Turkish Van breed and its phenotype, several small sized candidate regions were identified, most notably on Chr. E3 (Fig 3c) with *CLCN7* as a candidate gene for the hypopigmented appearance of the “Van” coat color [60]. By contrast, much fewer and considerably larger candidate regions were localized for the Bengal breed and two signifying a recent selection on Chrs.B4 (~ 5.5 Mb) and D2 (1.75 Mb). A focus on the candidate region on Chr. D2, which had a manageable number of genes, suggested *FGFR2* as a candidate gene for the softness of the Bengal’s coat [61].

The effect of the mode of inheritance of the breed-defining phenotype also has effects on the number and size of candidate regions. As an illustration, we refer to the regions of the Scottish Fold breed, which is defined by the dominantly-inherited folded ears [38] and compare it to American Curl, which characterized by the recessively-inherited backward curled ears (Fig 3a) [62]. Using two separate populations of the Scottish Fold breed (SFOLD1 and 2), we observed: (1) an averaged maximum candidate region size of 2 Mb, (2) significantly low number of shared windows between the two populations (S4 Table), and (3) lack of any candidate windows near the phenotype causing gene, *TRPV1* on Chr.D3. Conversely, the America Curl breed exhibited larger candidate regions two of which are indicative of selection of a recessive trait (Fig 3a). The largest candidate region (3.75 Mb) was located on Chr.E1 and among the 123 genes in the region, *MCRIP2* represents a likely candidate for curled ears. *MCRIP2* is a paralog of *FGFRL1*, which high localized expression of in cartilaginous tissue [63, 64]. A second candidate region also possessed a candidate gene for the curled ears, *PBX1*, and is implicated with ear malformations [65].

Lastly, breeding practices during the formation of a cat breed has apparent manifestations on the candidate regions under selection. We use the Abyssinian breed as an example of high inbreeding and the Bombay as an outcrossed breed. While Abyssinian is known to be an inbred breed [16, 44, 55], Bombay is documented to be a hybrid breed between the Eastern Burmese and the Western American Shorthair (Fig 1, S1 Table). The signs of inbreeding of Abyssinian contributed to eight candidate regions with a combined size of 15.5 Mb whereas the outcrossing of Bombay allowed detecting as two candidate regions with a sum of 2.5 Mb.

### Other candidate regions and candidate genes

We examined the robustness of our analysis using known phenotype-related genes and inspected their colocalization in candidate regions under selection (e.g. Cornish Rex [46], Devon Rex [32], Abyssinian [25], Siamese [22], and in longhaired breeds [66, 67]). However, beyond the aforementioned known breed-defining variants, we suggested a number of candidate genes that resided in identified regions of different breeds and might be responsible for phenotypes with still unknown causative genes (Table 1).

The Maine Coon breed is known for its large size (Fig 3b) [68] and its candidate region on Chr.A1 might be related to the large body size. The four genes within the region, *GABRG2*, *GABRA1*, *GABRA6*, and *GABRB2*, are known to alter the tastebuds and enhance food craving [69, 70]. On the other extreme of body-form, we have detected a shared candidate region across Siamese, Oriental, and Peterbald on Chr.D3. The three Eastern breeds exhibit thin-tubular body (Fig 4). The shared candidate region highlighted *ADGRD1* as a potential candidate gene for its association with metabolism and body-weight [71, 72].

Although several genes responsible for the curly coat in cat breeds have been identified [32, 33, 35, 46], the genetic variant responsible for the curled coat of LaPerm is still unknown. We have identified 3.75 Mb candidate region in LaPerm with likely involvement of EPGN and EGER to the unique curly coat of the breed [73]. Examining the Birman via its two populations and the highly shared candidate windows showed several windows on Chr.B1 where *KIT* is located. The white coat that is located on Birman cats’ feet and referred to as “gloves”, has been linked to *KIT* (Fig 4) [18]. However, a large candidate region was observed on Chr.B4 with *KITLG* as a potential second player in the formation of the “gloves” phenotype.

The single largest candidate region across all breeds was observed in Ragdoll on Chr.A1 (7 Mb) and shared among its two populations (RAG1 and 2). Even though the region contained forty genes, the *Lysosomal Associated Membrane protein 1 (LAMP1)* represented a candidate gene that may explain multiple aspects related to Ragdoll cats. *LAMP1* is associated with *Lysosomal Trafficking Regulator* (*LYST*), which causes the multi-system Chediak-Higashi Syndrome. The syndrome manifests as dilution of skin, hair, and iris colors, bleeding diathesis, and recurrent infections (reviewed in [74]). Although the *LYST* and its severe consequences have been identified and described in cats (cited in [75]), we suspect that the large candidate region on Chr.A1 and *LAMP1* more moderately contributes to the phenotypic characteristics of Ragdoll cats (e.g. light coat and eye colors) and explains its mucopolysaccharidoses [76].

We have empirically identified candidate regions under selection in twenty-six cat breeds and due to the nature of the data (i.e., Array SNPs ascertained from different breeds), it is not possible to rigorously account for and distinguish between the effects of demography and population structure from artificial selection. Using whole-genome sequence data can foreseeably provide more definitive confirmations of the candidate regions presented. Furthermore, our suggested candidate genes also await an in-depth molecular investigation to reach satisfying conclusions.

## Materials and methods

### Ethics statement

This study does not require an ethics statement.

### SNP genotype dataset acquisition

A previously published SNP dataset using the Illumina Feline-infinium 63K SNP array was used in the current study [55]. The dataset contains 34 domestic cat breeds (n = 1,570). A subset of the genotype dataset was selected by excluding cat breeds with sample size less than 10 individuals, and samples with genotyping rate less than 90%. Sample genotyping rate was determined by using the command (--mind) in plink v1.7 [77] (S1 and S2 Data).

Within each cat breed, only unrelated samples were retained to perform further analyses. The genetic relatedness between individuals within each breed was determined using the statistic (PI_HAT) that measures the proportion of Identity by descent (IBD) of alleles for pairs of individuals and was calculated by the command (--genome) in plink v1.7 [77]. A (PI_HAT) of 0.25 or lower was used to select samples that were unrelated at least to the grandparent level. The final list of samples included in the study and their corresponding breed assignment are shown in S5 Table.

### SNP genotyping and quality control

The original datasets consists of ~63K SNP markers [55] and the following markers were excluded from downstream analyses: (1) X-chromosome SNPs, (2) unmapped SNPs, (3) SNPs with genotyping rate less than 90%, and (4) SNPs unmapped to felCat 9.0 [51]. The final list of SNPs included in the study and their updated positions to felCat 9.0 are shown in S6 Table. SNP genotyping rate was determined using (--geno) while percentage of monomorphic SNPs was determined with (--freq) both implemented in plink v1.7 [77].

For each population independently, the following summary statistics were calculated using PLINK v1.7 [77]: (1) the function (--freq) was used to calculate the mean and standard deviation of minor allele frequency (MAF), (2) the mean and standard deviation of observed and expected heterozygosity were obtained using the function (--hardy), and (3) the mean individual inbreeding coefficient (F) in each breed was determined with the command (--het).

### Population structure

Population structure and relationships between different cat breeds (n = 26) were analyzed using Principal Component Analysis (PCA) that is based on the variance-standardized relationship matrix implemented in PLINK v1.9 [78]. With the default setting, (--pca) function was used to generate the first 20 principle components. The first two components of the PCA (PC1, PC2) were used to illustrate the overall population structure of the breeds.

### F_st_ and *d*_*i*_ calculations

F_st_ was calculated following Weir and Cockerham method [79], which provides accurate estimates of F_st_ especially when sample sizes are uneven across breeds or are small [80] and using vcftools v 0.1.13 [81]. Original files (.ped and .map) were first converted into Variant Call Format (vcf) file using (--recode-vcf) command in plink v1.9 [78]. Using vcf files and (--weir-fst-pop) command of vcftools, F_st_ was calculated pairwise between all breeds and for each SNP.

A simple summary statistic (*d*_*i*_) that measure locus specific divergence in allele frequencies was previously developed and implemented [15, 46, 48]. A script written in R was used to calculate *d*_*i*_ values according to the equation for each breed independently against all breeds. At each SNP, the *di* represents the sum of the values calculated above.

### Candidate regions under selection

Based on SNP distribution and ensure sufficient SNP density in our scans, each chromosome was divided into 500Kb overlapping windows (e.g., window1: 0-500Kb, window2: 250-750Kb). The *di* estimates were averaged for all SNPs within each window (n = 9108 windows). The *di* averaged values of each window within each chromosome were visualized via an R Manhattan plot script. The 99^th^ percentile was used as a threshold for window prioritization and the top 92 windows were considered candidate windows under selection. To avoid false positives and to allow comparison across breed beyond the 99^th^ percentile threshold, the candidate windows were further categorized into candidate regions if three or more windows overlapped. The resulting candidate regions were visualized across breeds in chromosomal plots based on the latest cat genome assembly (felCat 9.0 [51]).

## Supporting information

S1 Fig Chromosomal representation of candidate windows under selection across cat breeds. Each autosomal chromosome is plotted separately. Chromosomes are positioned on the x-axis with gray thin lines representing SNP position. Each breed is represented on the y-axis and traced by a dotted horizontal line for ease of reference. Western, “Middle”, and Eastern, are colored red, yellow, and blue, respectively. Individual colored-bars corresponds to a 500 Kb candidate window (92 for each breed). Overlapping windows are displayed as darker colored bars and combined are considered candidate region under selection whereas individual scattered are of lightly colored and dismissed from gene content survey. Vertical dashed lines are placed according to the genomic positions (felCat9) of known genetic disease/phenotype implicated genes in cats. Boxed candidate regions were chosen as representative candidate regions in individual breeds or in multiple breeds and further investigated for candidate genes.
(PDF)

S2 Fig Proportions of candidate windows under selection categorized into four classes based on number of overlapping windows. (TIFF)

S3 Fig Summary of candidate regions under selection for all cat breeds combined. (a) Overview of the conservative number of candidate regions per chromosome. (b) Number of candidate regions in relation to the chromosome size. Chr. B4 and Chr. E3 deviates from linear relationship between chromosome size and number of candidate regions. (c) Number of overlapping windows and (d) size (Mb) per candidate region. The majority of candidate regions are composed of three overlapping widows and 1Mb size.
(TIFF)

S4 Fig A comparison of the combined size (Mb) of candidate regions under selection across cat breeds. (TIFF)

S1 Table Summary statistics of investigated cat breeds. (XLSX)

S2 Table A List of candidate 500Kb windows under selection of 26 cat breeds. (XLSX)

S3 Table A List of candidate regions under selection including lists of genes within each candidate region for 26 cat breeds. (XLSX)

S4 Table An intra-specific comparison of candidate windows between two populations within a breed. (XLSX)

S5 Table A list of the samples used in the study. (XLSX)

S6 Table A list of the SNPs used in the study with updated positions to felCat 9.0. (XLSX)

S1 Data SNP genotype data used in the study (ped file). (ZIP)

S2 Data SNP positions of the genotype data used in the study (map file). (ZIP)
