# Supplementary figures and images for: Patterns of allele frequency differences among domestic cat breeds assessed by a 63K SNP array
Source: PLoS One. 2021 Feb 25;16(2):e0247092. doi: 10.1371/journal.pone.0247092 (PMC7906347; doi:10.1371/journal.pone.0247092)

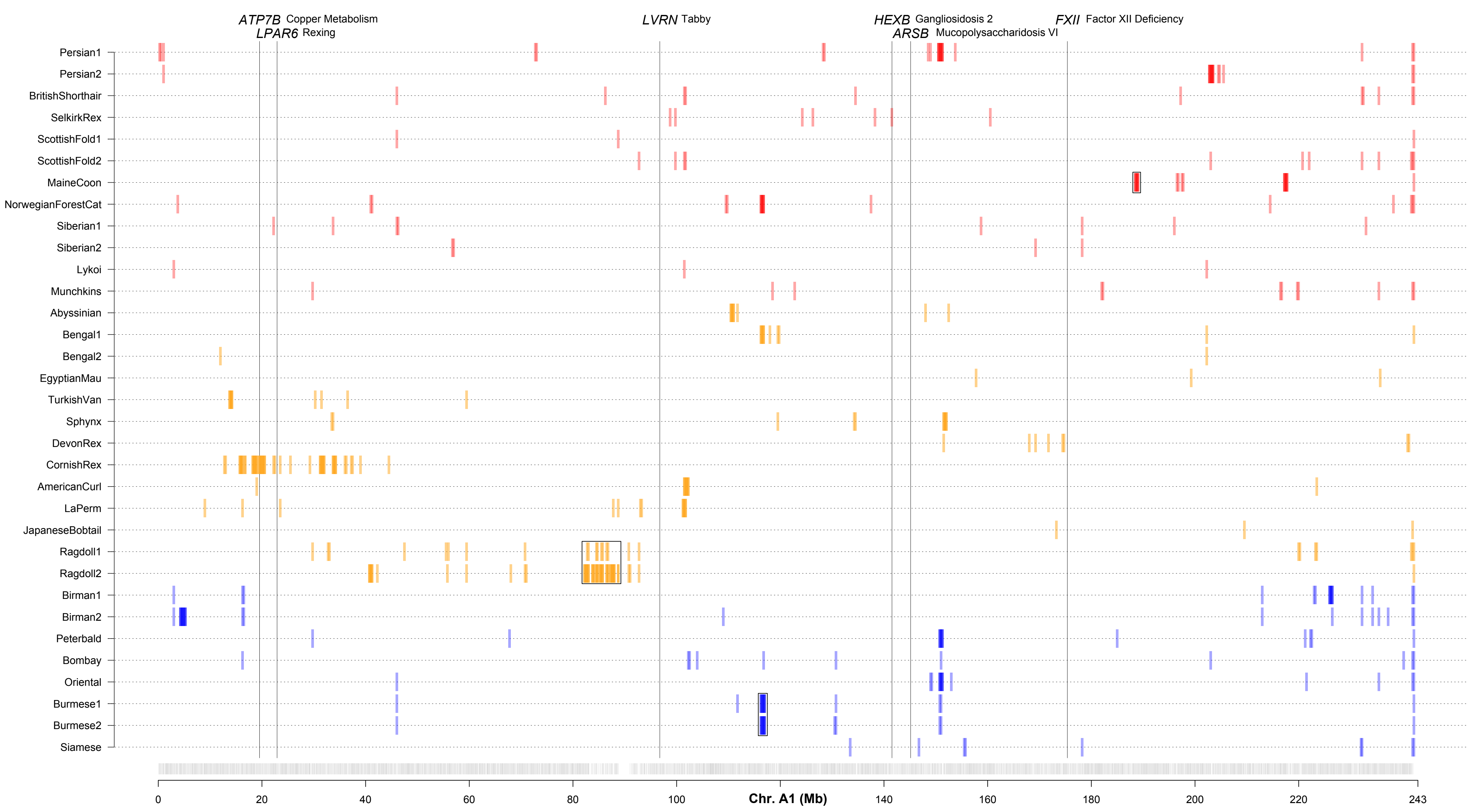

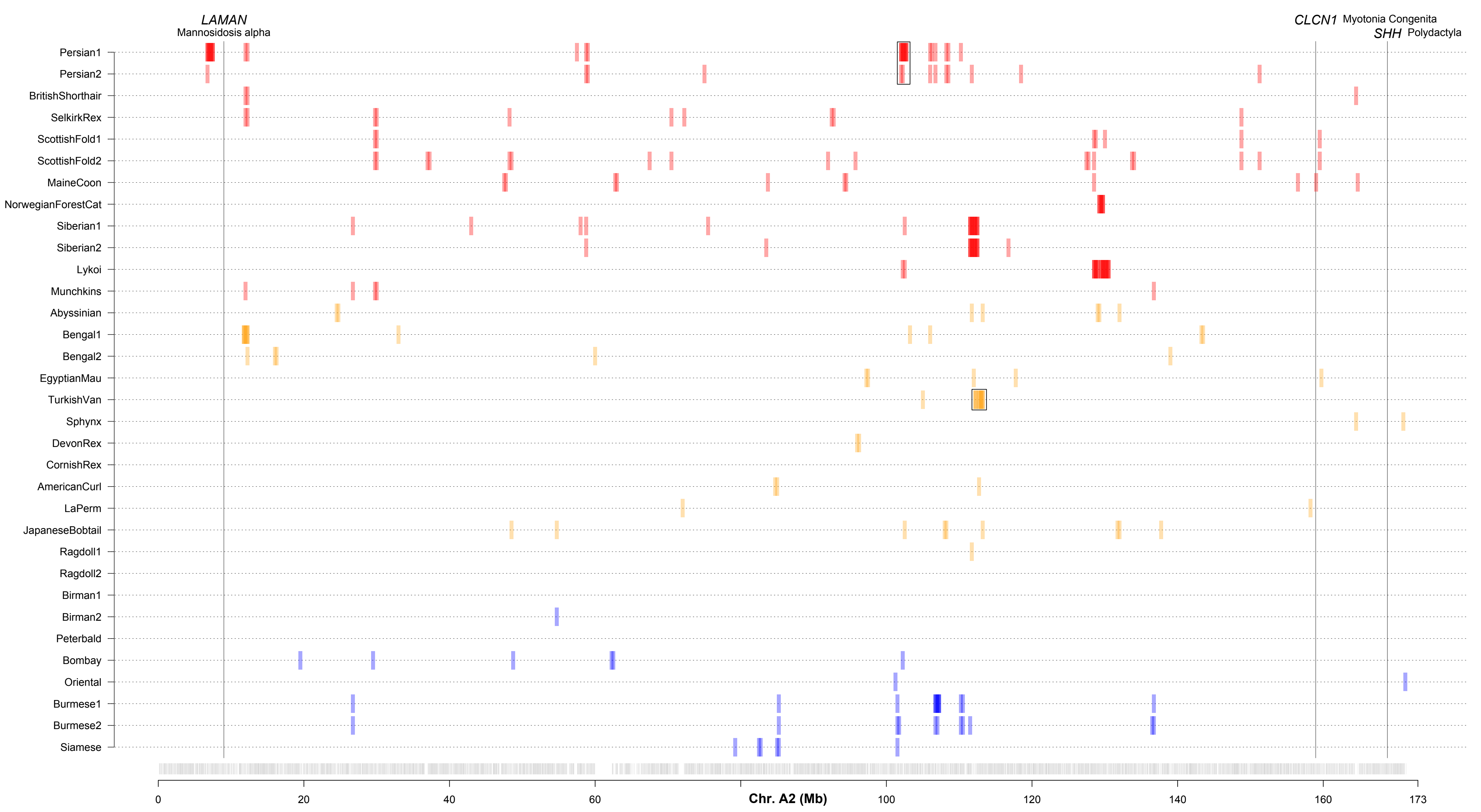

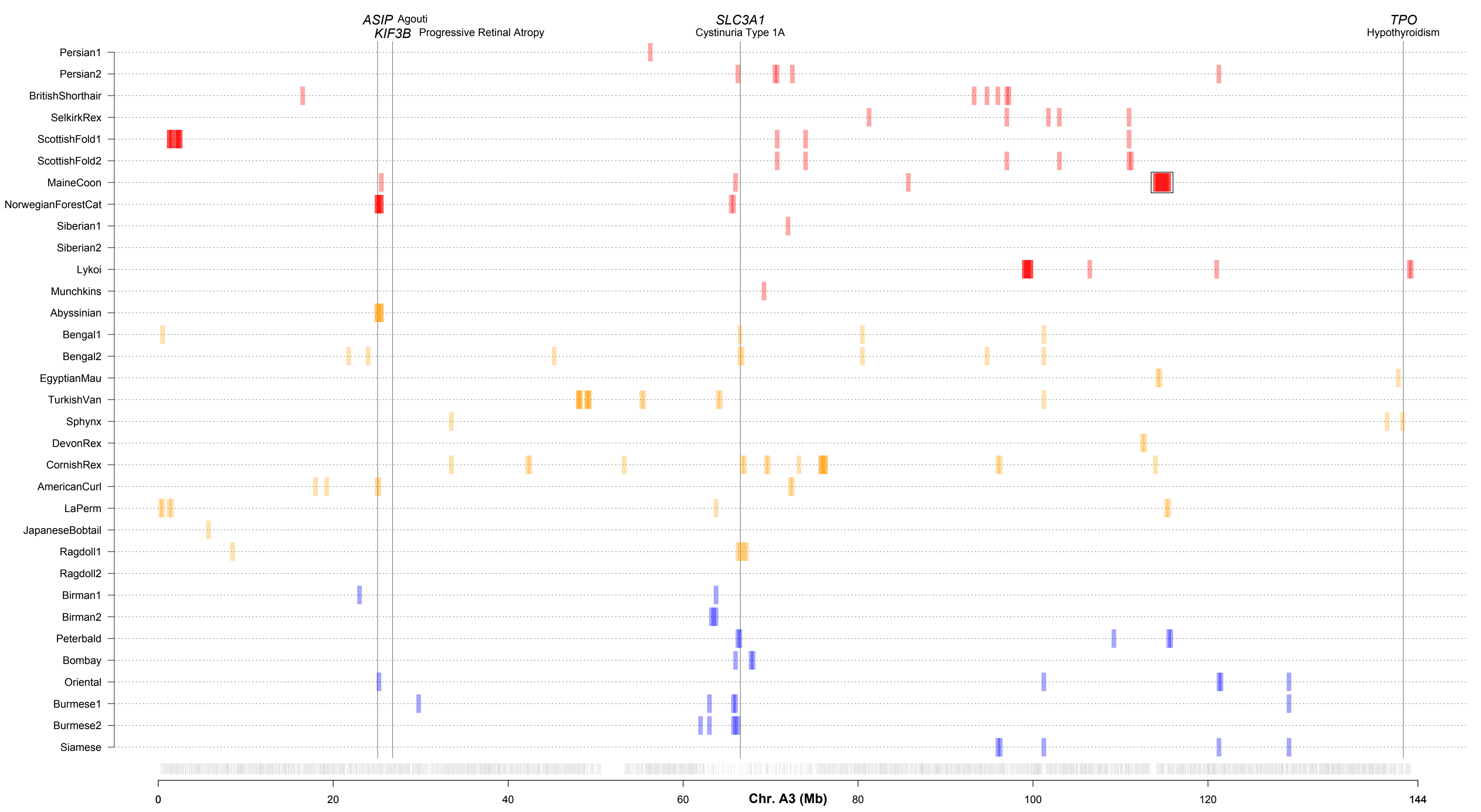

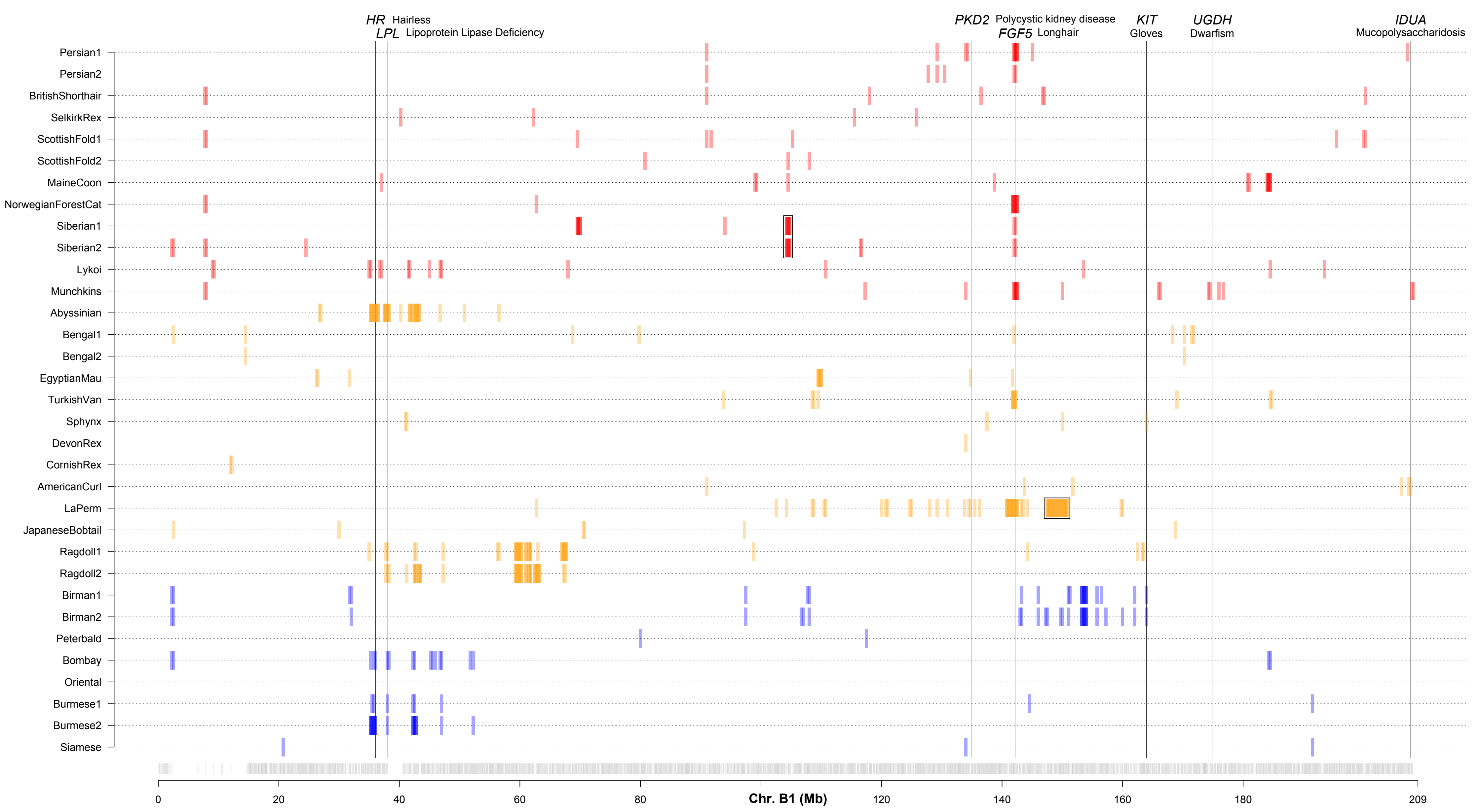

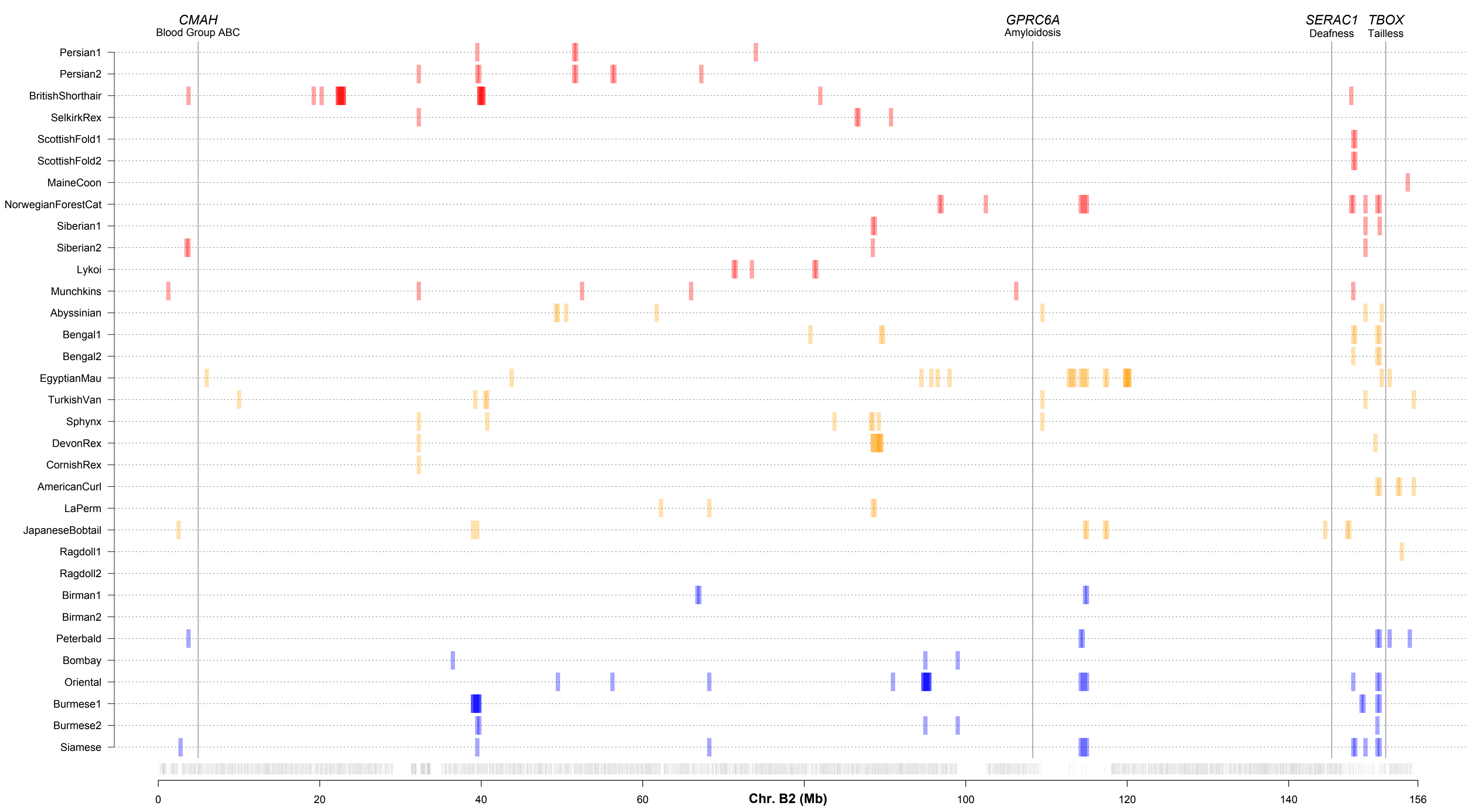

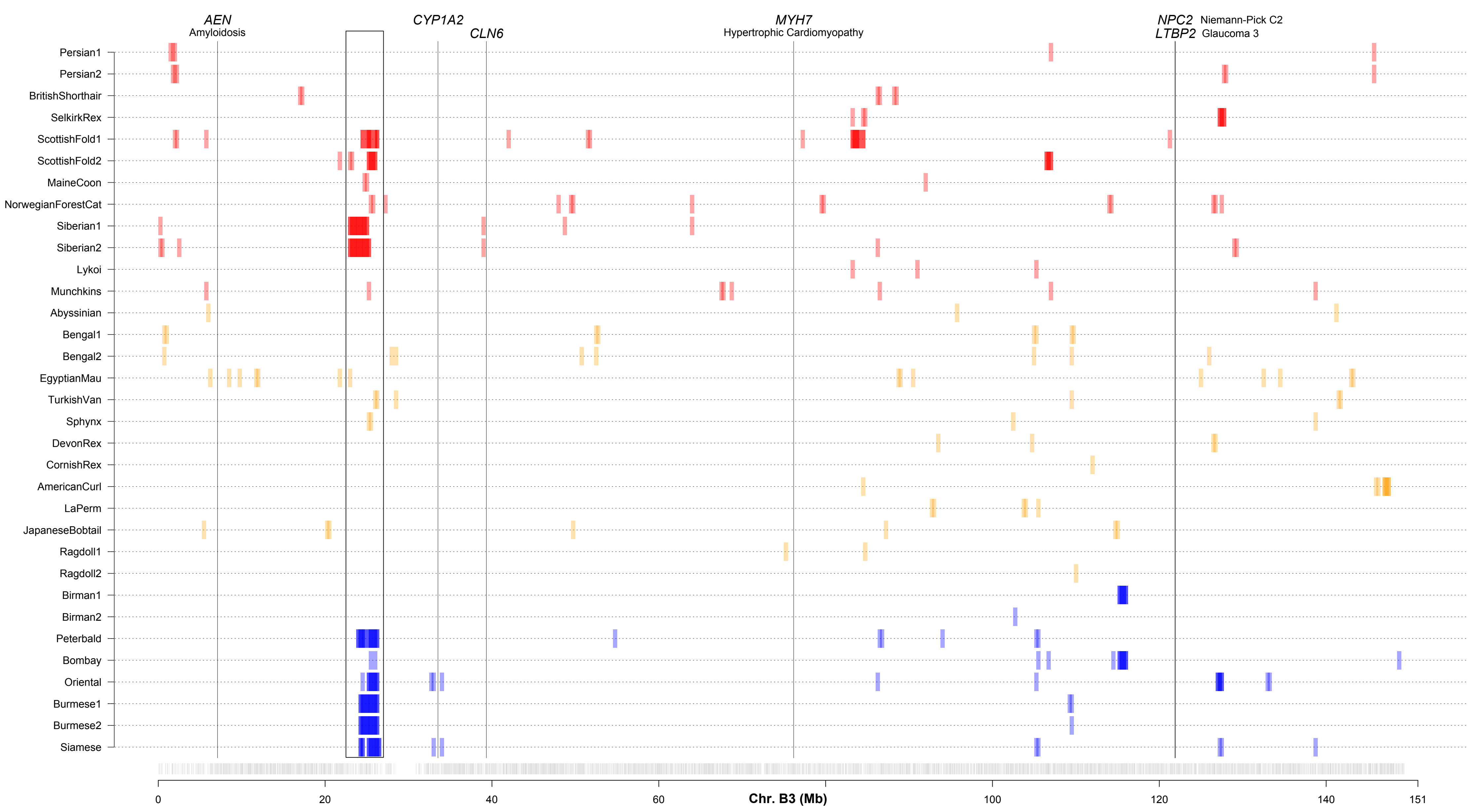

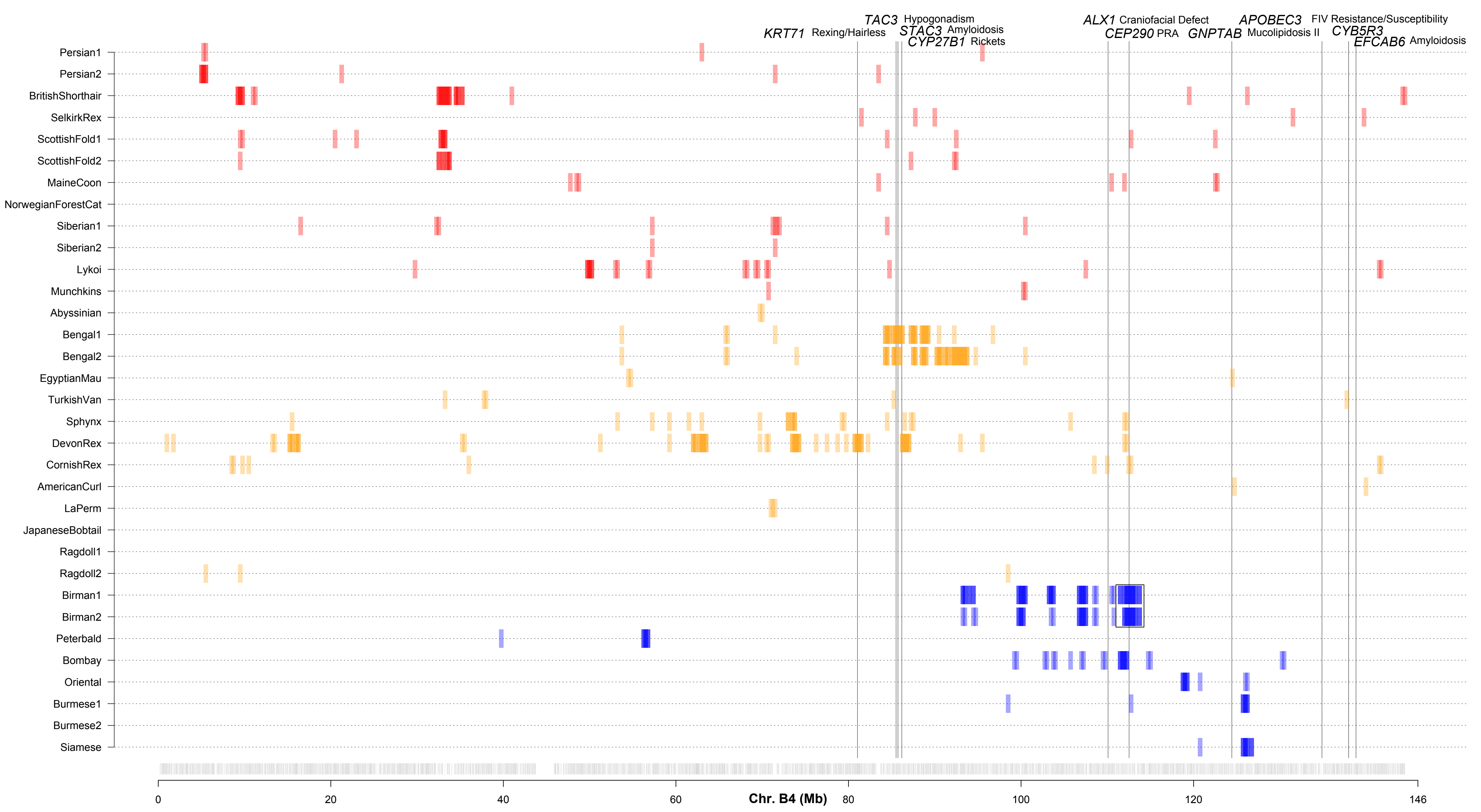

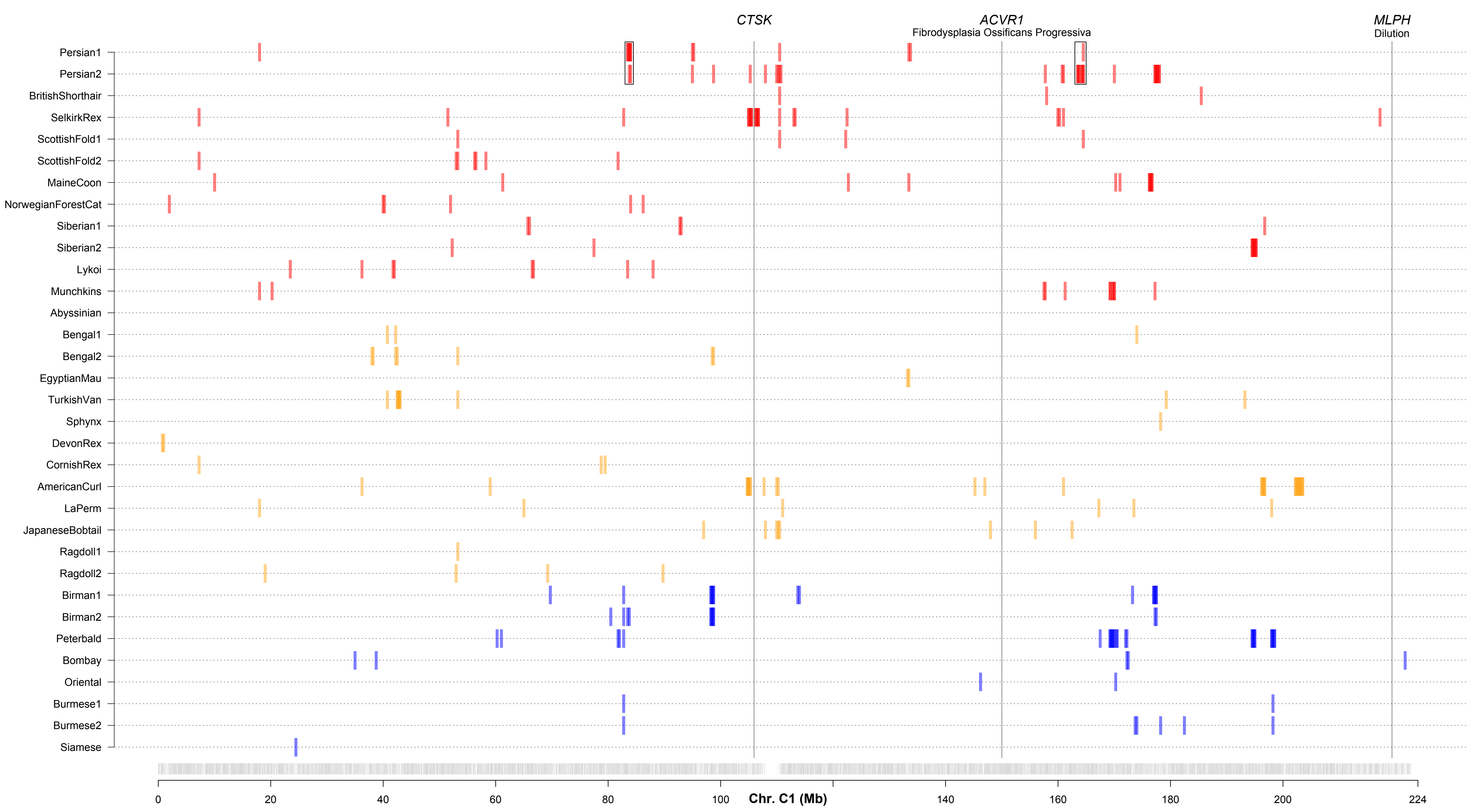

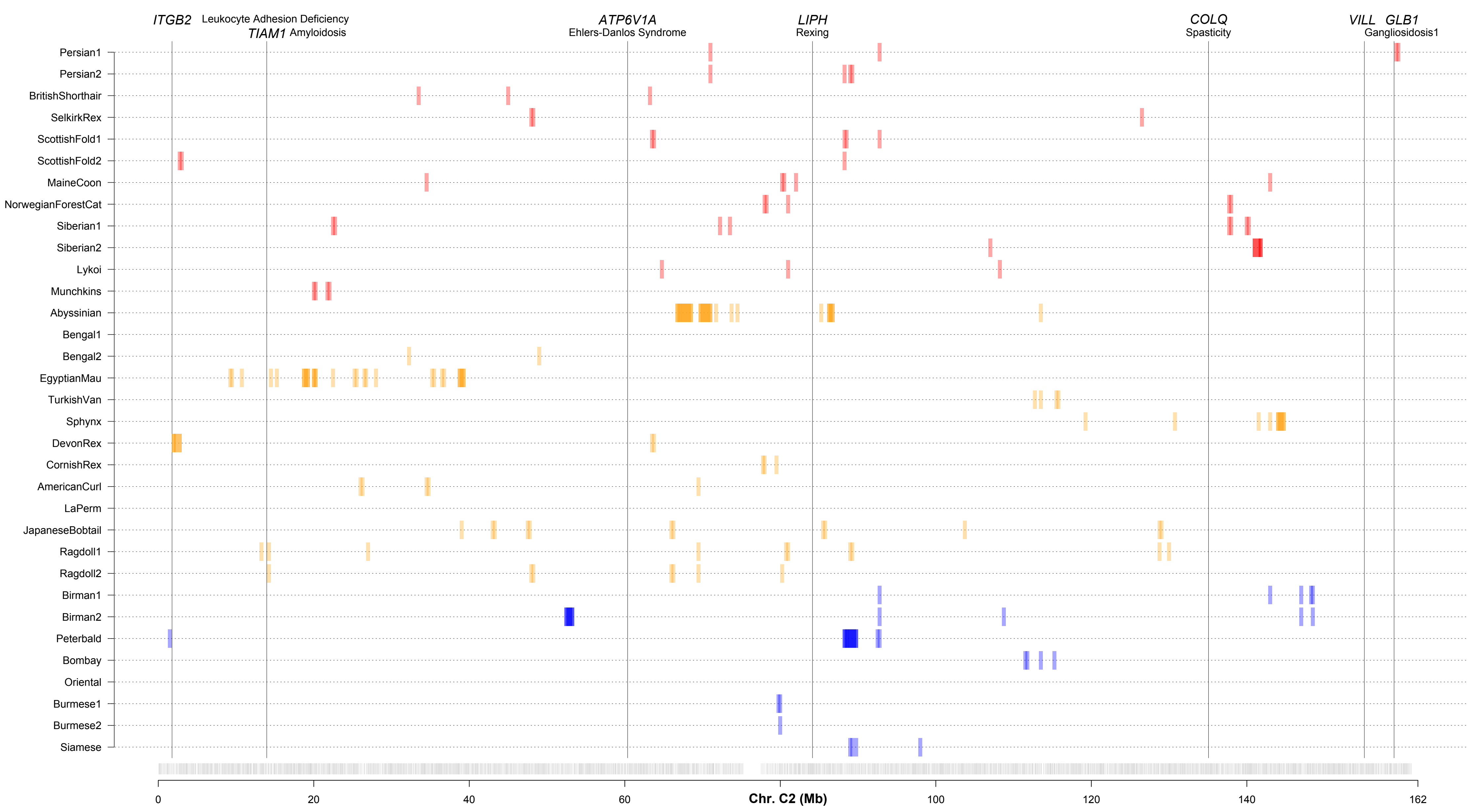

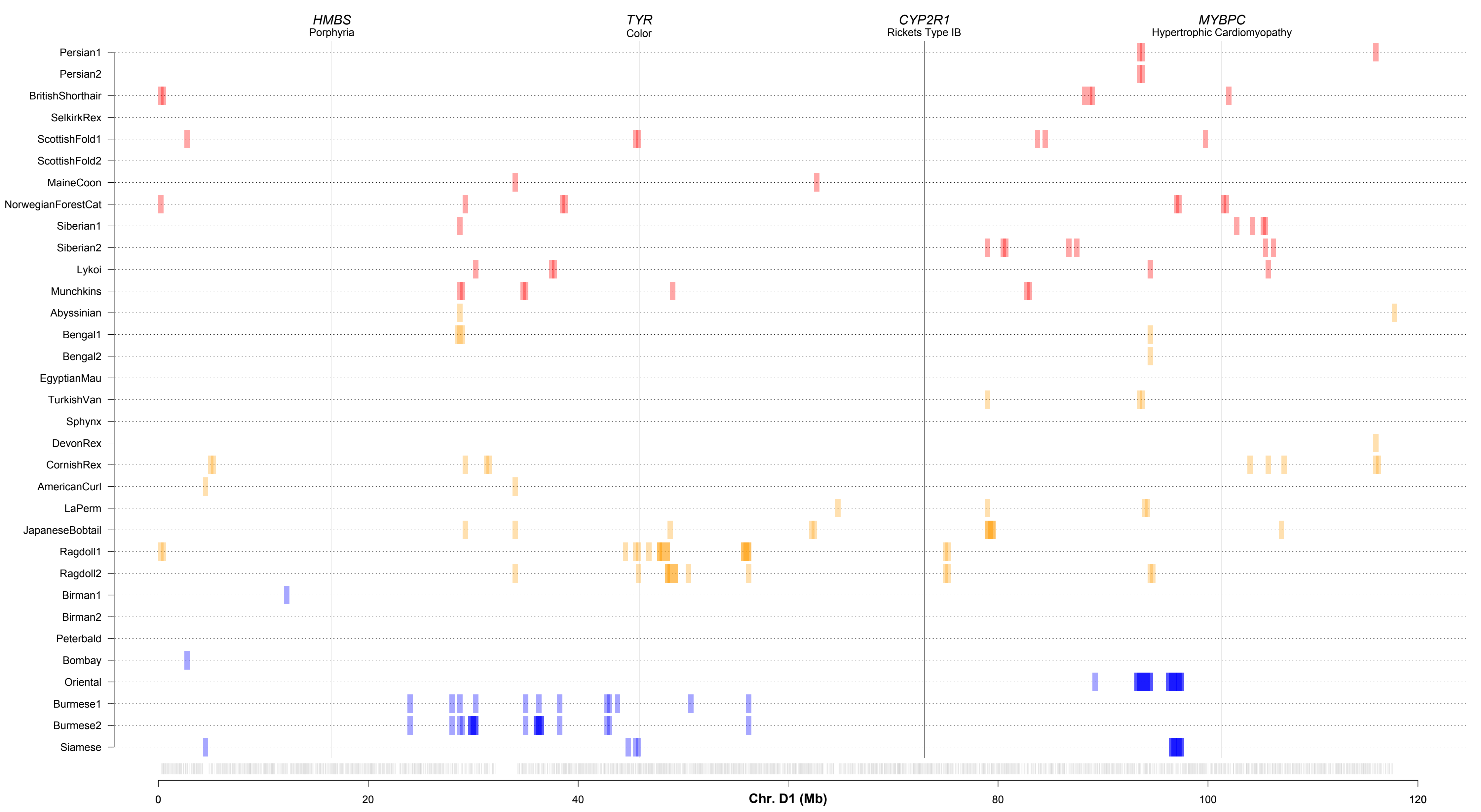

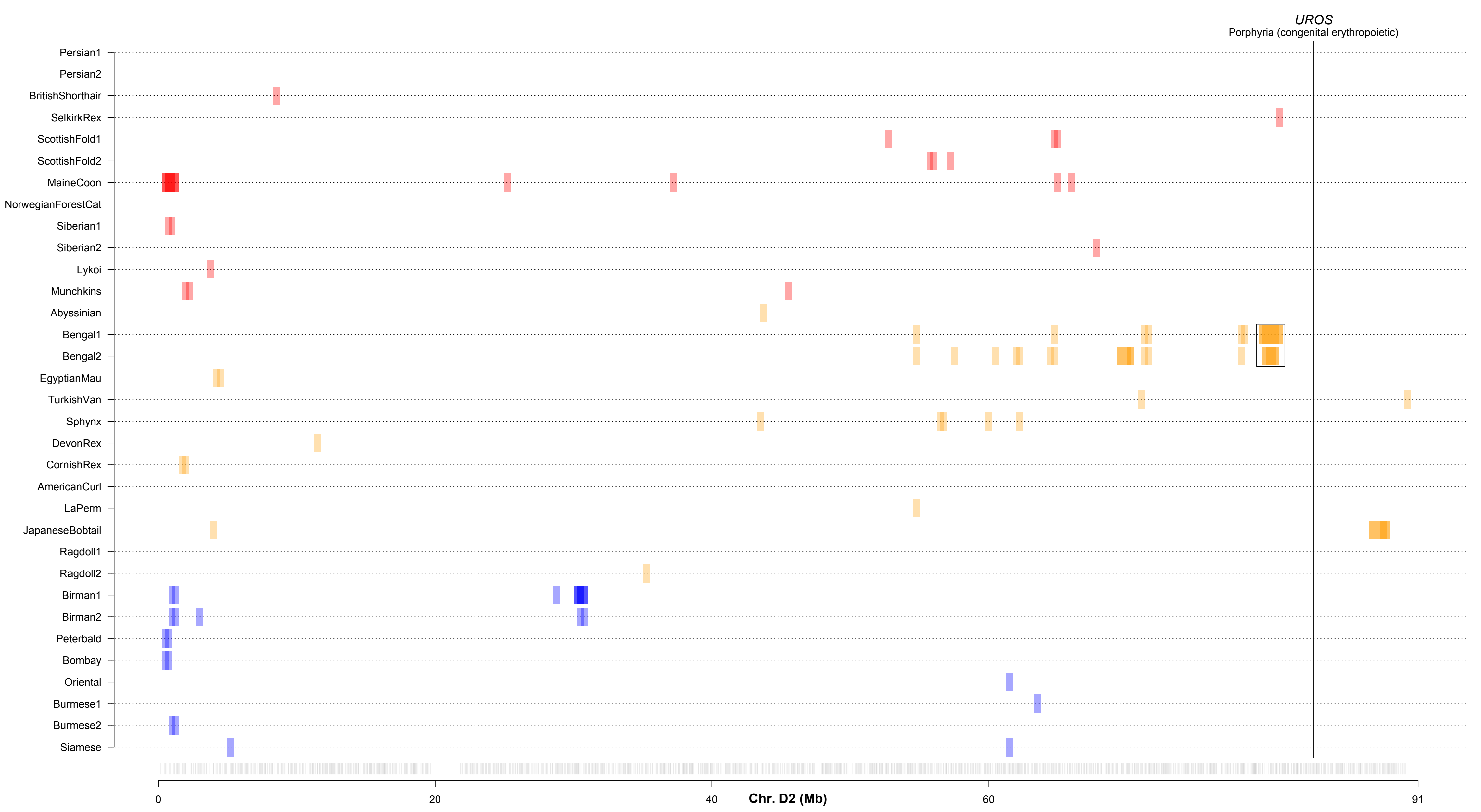

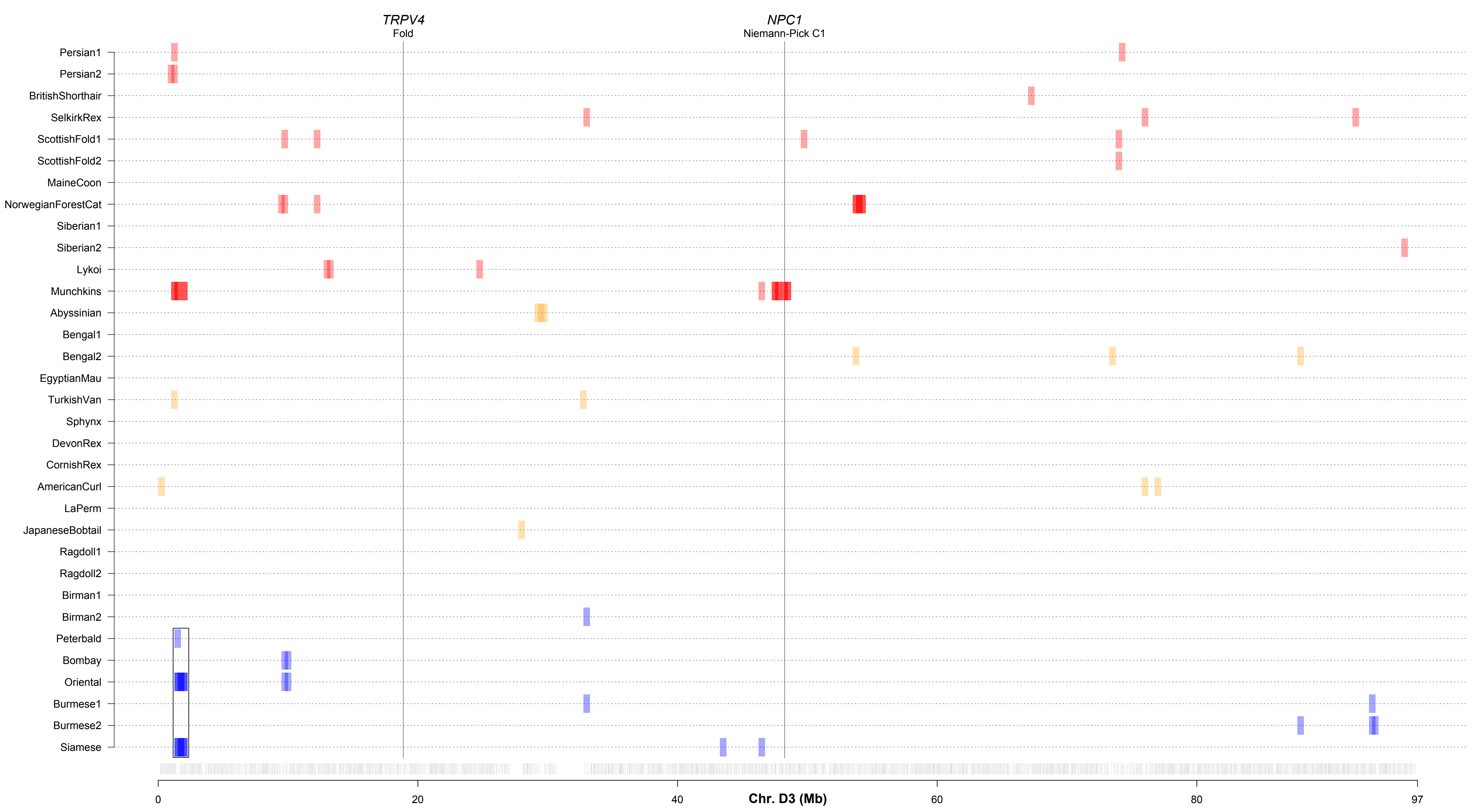

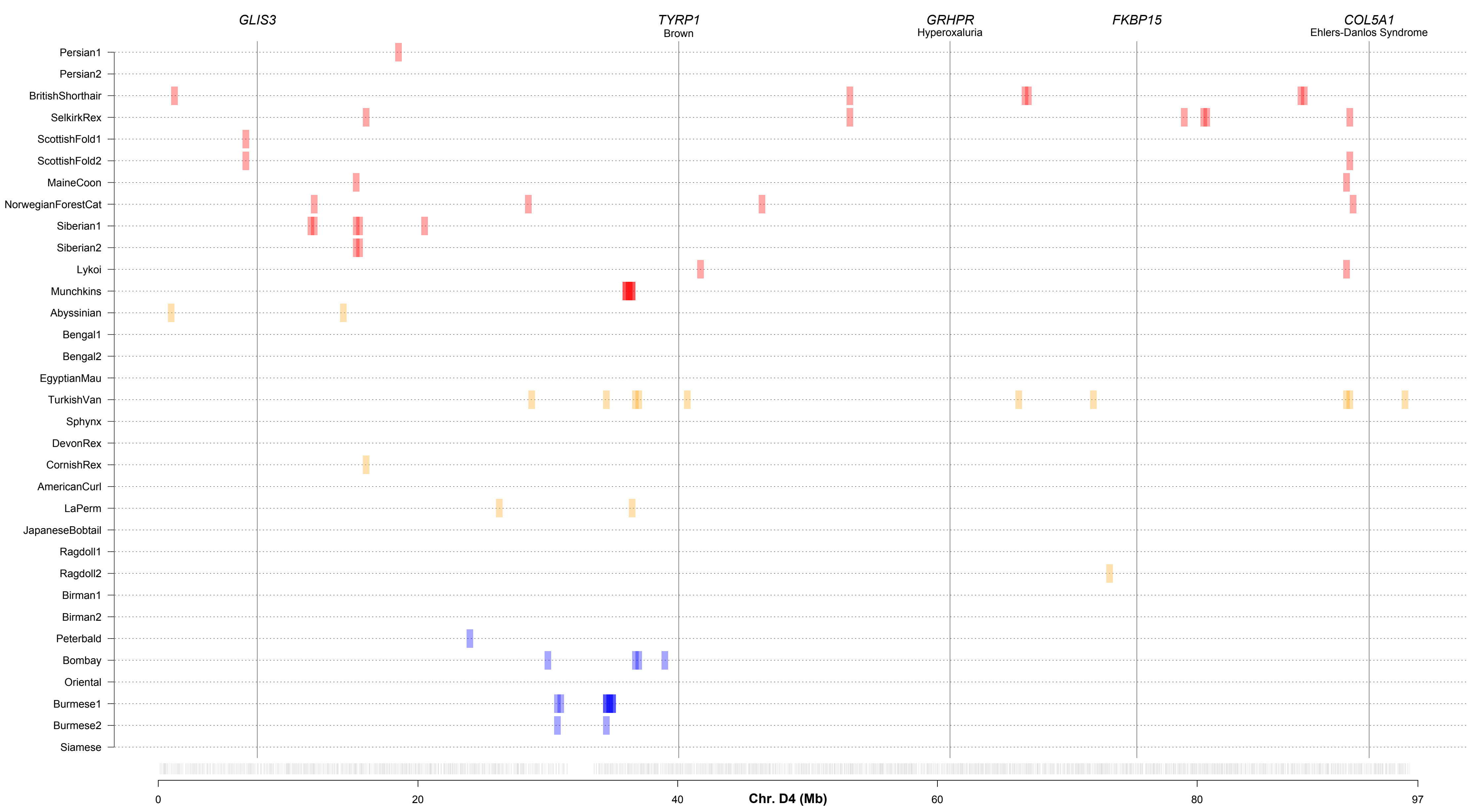

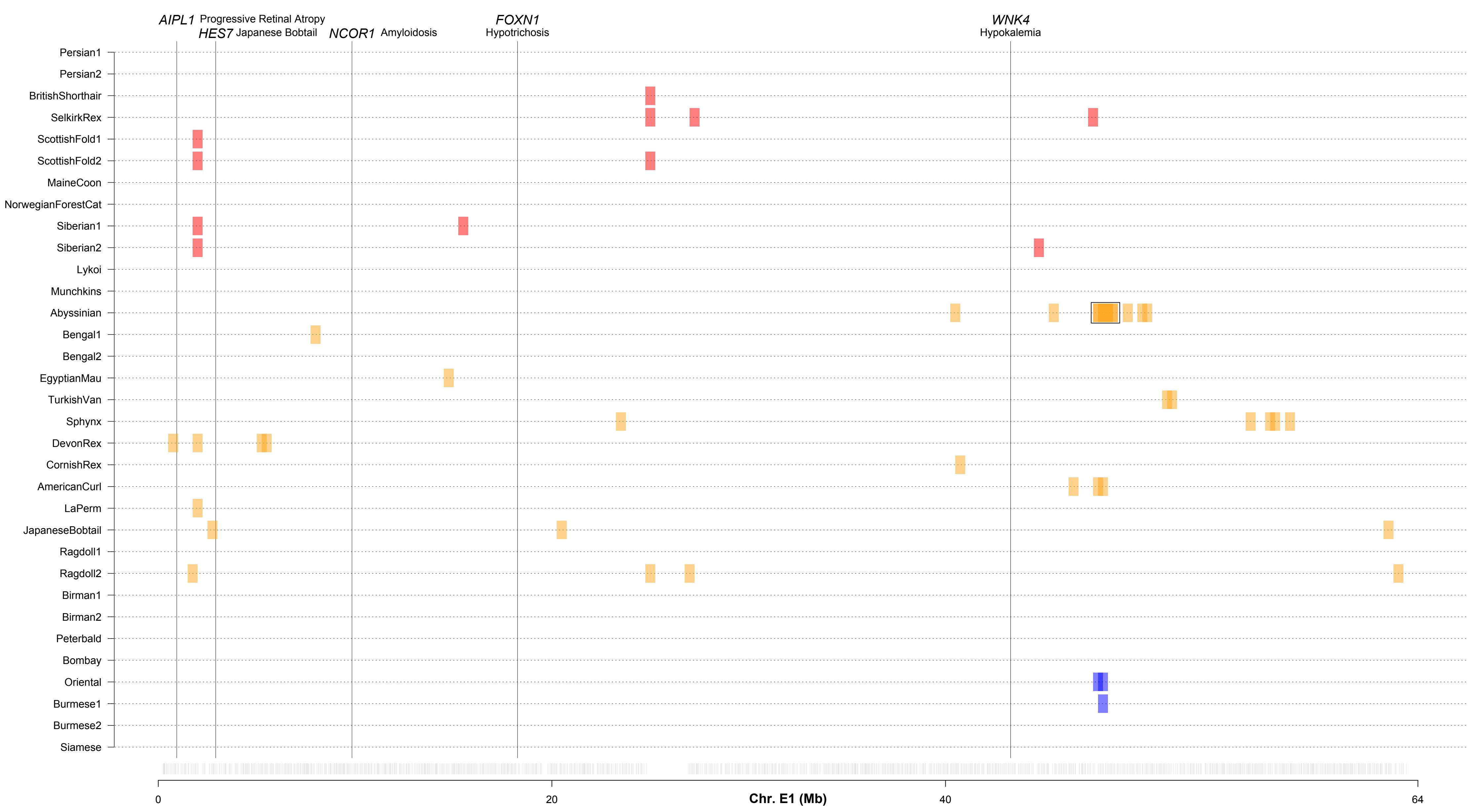

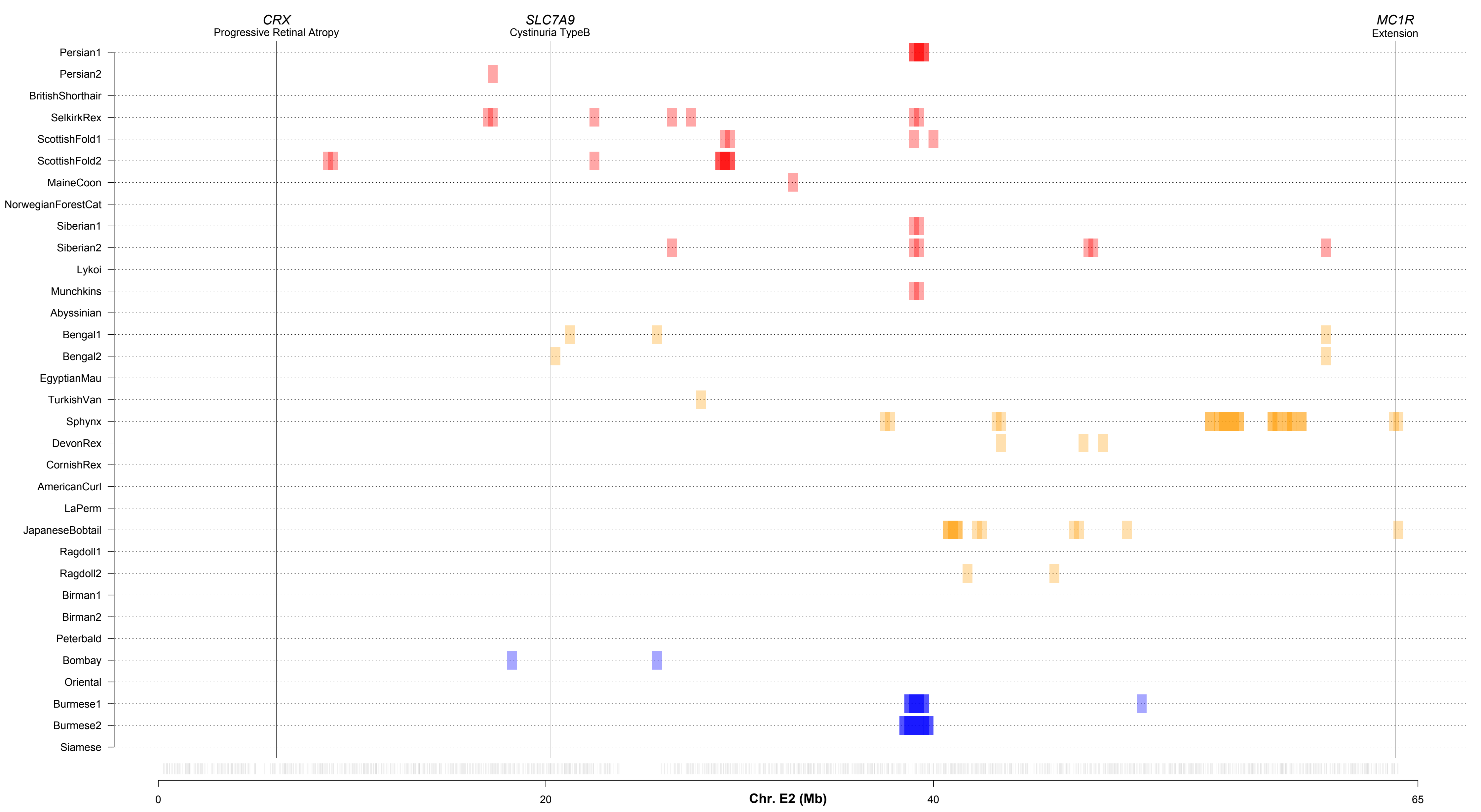

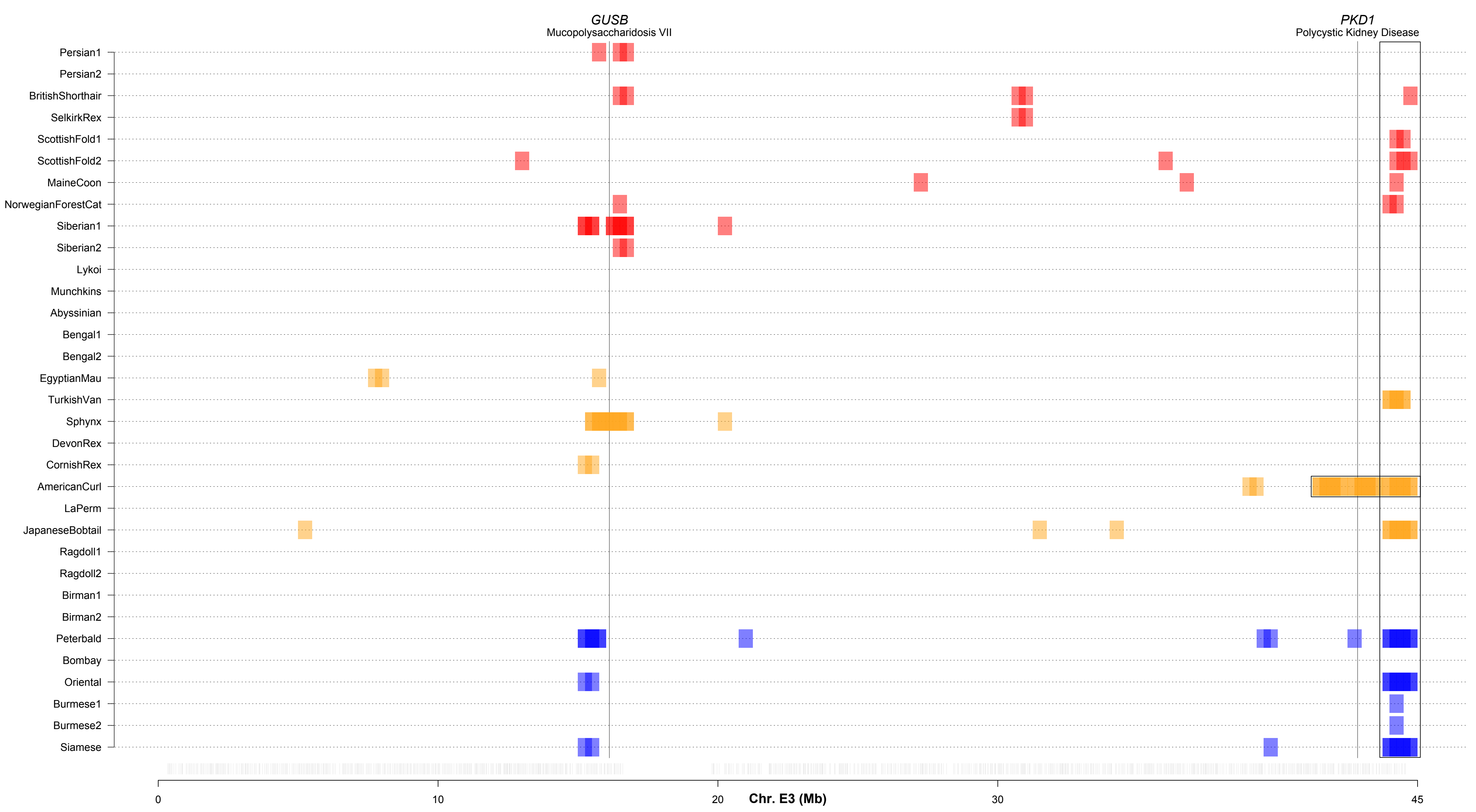

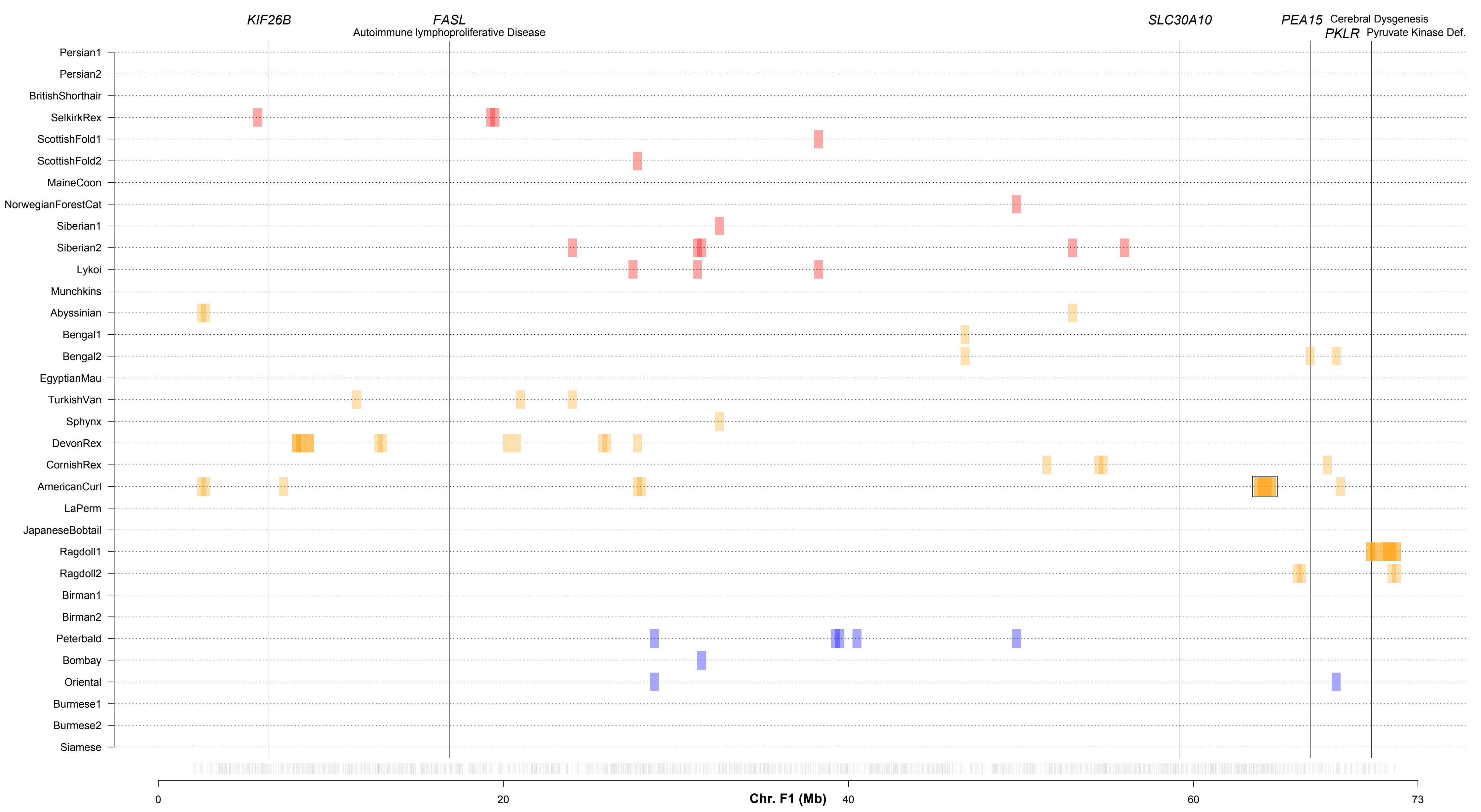

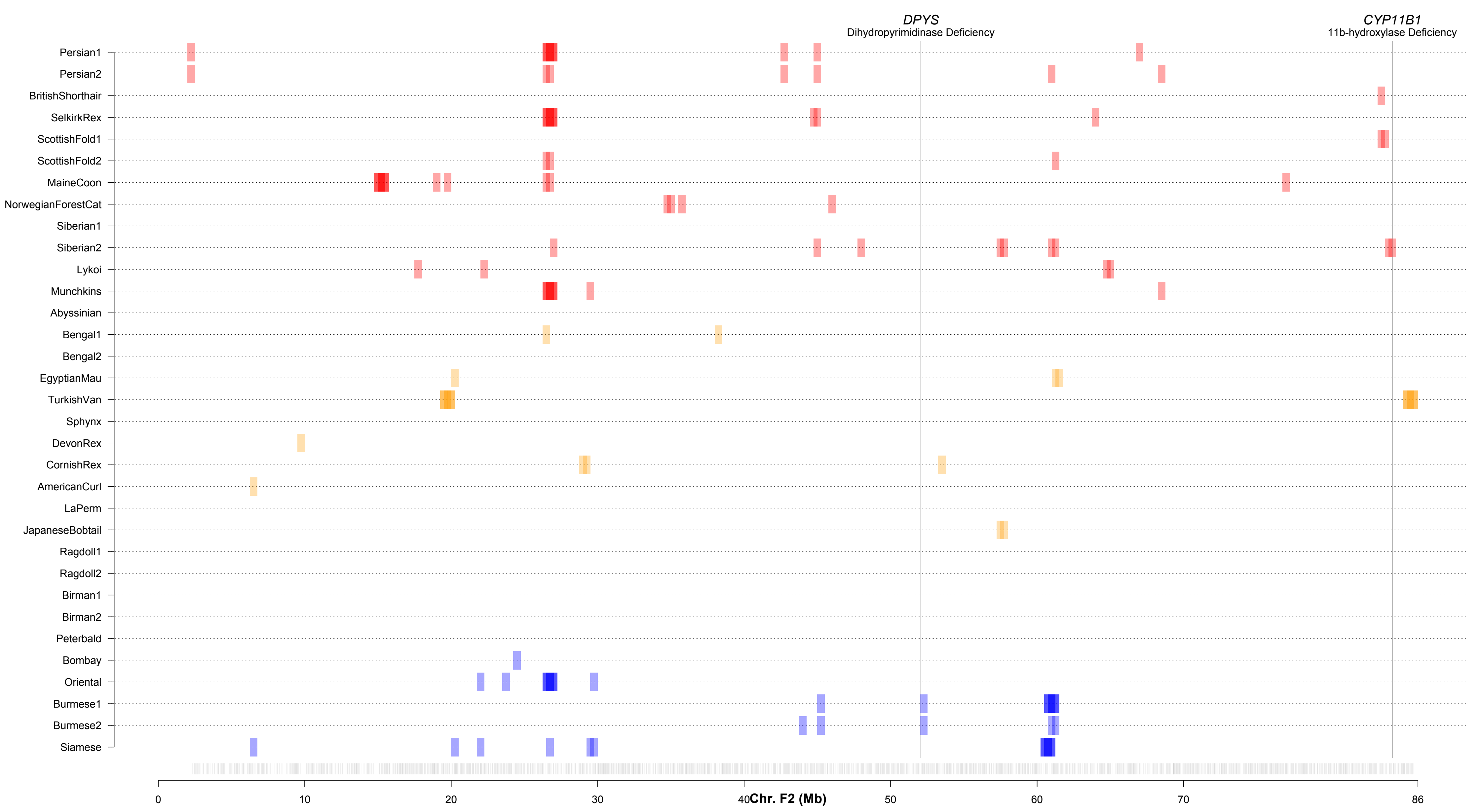

Supplement: S1 Fig — Each autosomal chromosome is plotted separately. Chromosomes are positioned on the x-axis with gray thin lines representing SNP position. Each breed is represented on the y-axis and traced by a dotted horizontal line for ease of reference. Western, “Middle”, and Eastern, are colored red, yellow, and blue, respectively. Individual colored-bars corresponds to a 500 Kb candidate window (92 for each breed). Overlapping windows are displayed as darker colored bars and combined are considered candidate region under selection whereas individual scattered are of lightly colored and dismissed from gene content survey. Vertical dashed lines are placed according to the genomic positions (felCat9) of known genetic disease/phenotype implicated genes in cats. Boxed candidate regions were chosen as representative candidate regions in individual breeds or in multiple breeds and further investigated for candidate genes. (PDF) [file pone.0247092.s001.pdf]

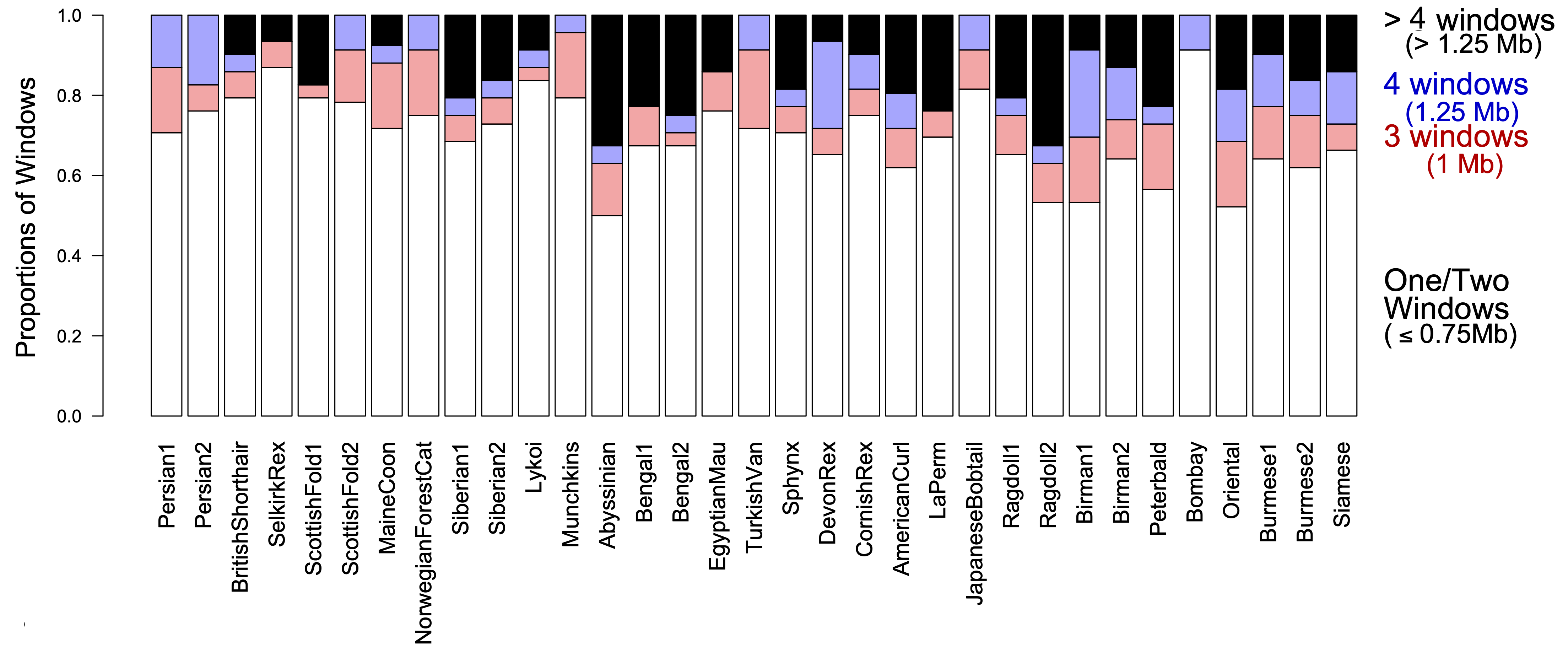

Supplement: S2 Fig — (TIFF) [file pone.0247092.s002.tiff]

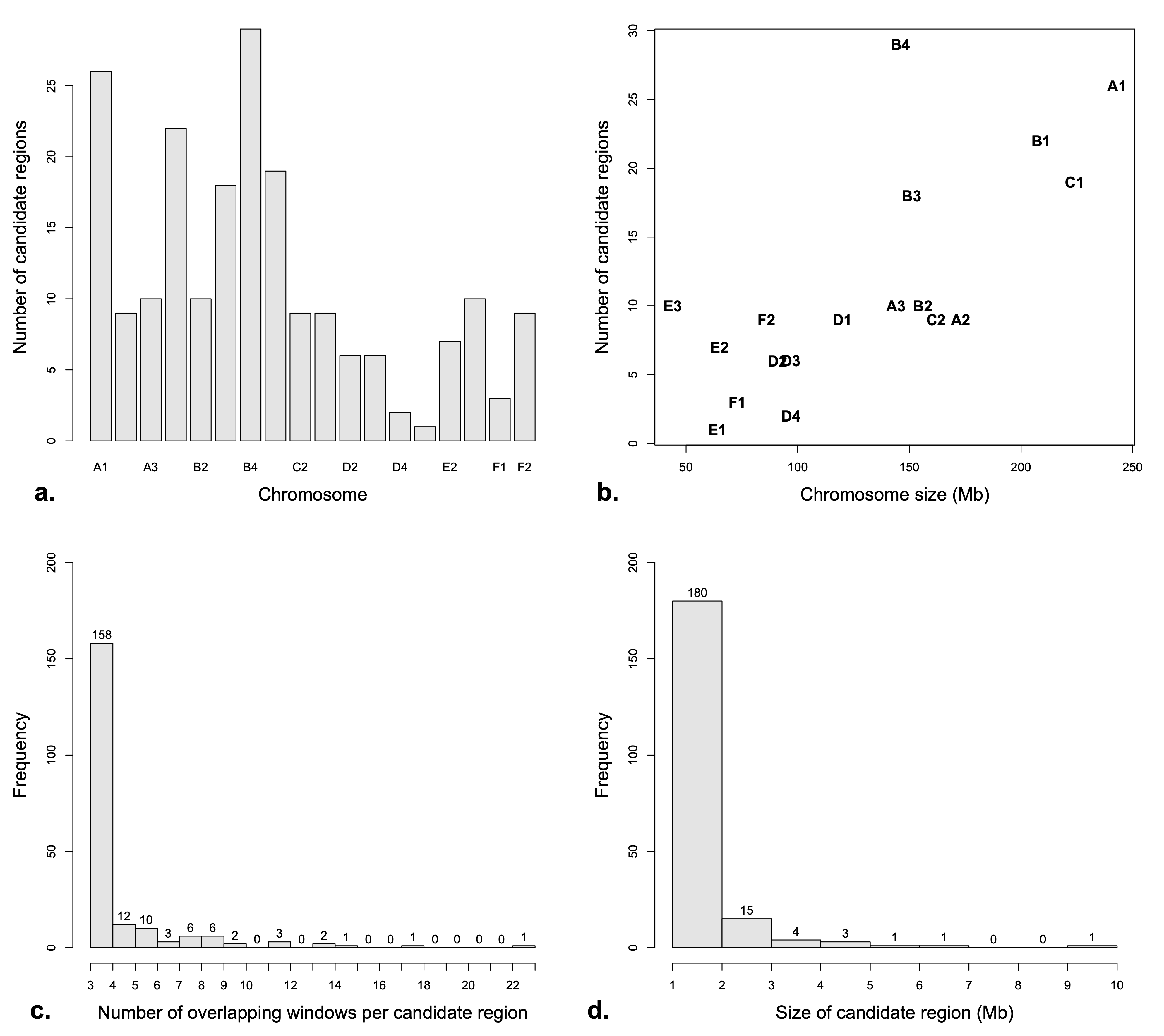

Supplement: S3 Fig — (a) Overview of the conservative number of candidate regions per chromosome. (b) Number of candidate regions in relation to the chromosome size. Chr. B4 and Chr. E3 deviates from linear relationship between chromosome size and number of candidate regions. (c) Number of overlapping windows and (d) size (Mb) per candidate region. The majority of candidate regions are composed of three overlapping widows and 1Mb size. (TIFF) [file pone.0247092.s003.tiff]

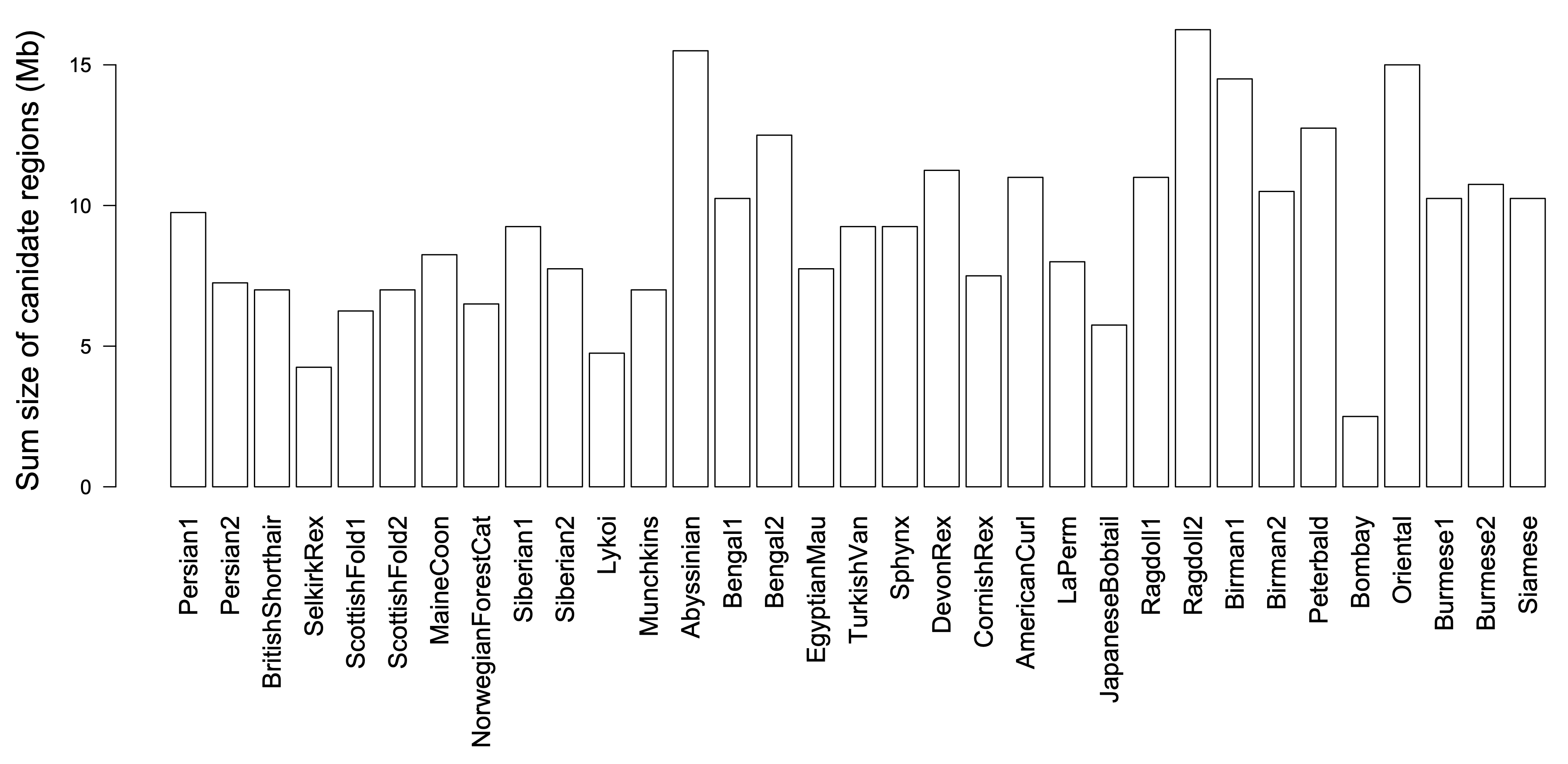

Supplement: S4 Fig — (TIFF) [file pone.0247092.s004.tiff]
